# Balance between competing spectral states in subthalamic nucleus is linked to motor impairment in Parkinson’s disease

**DOI:** 10.1093/brain/awab264

**Published:** 2022-03-29

**Authors:** Saed Khawaldeh, Gerd Tinkhauser, Flavie Torrecillos, Shenghong He, Thomas Foltynie, Patricia Limousin, Ludvic Zrinzo, Ashwini Oswal, Andrew J. Quinn, Diego Vidaurre, Huiling Tan, Vladimir Litvak, Andrea Kühn, Mark Woolrich, Peter Brown

**Affiliations:** 1 MRC Brain Network Dynamics Unit, University of Oxford, Oxford OX1 3TH, UK; 2 Nuffield Department of Clinical Neurosciences, University of Oxford, Oxford OX3 9DU, UK; 3 Oxford Centre for Human Brain Activity, Wellcome Centre for Integrative Neuroimaging, University of Oxford, Oxford OX3 7JX, UK; 4 Department of Neurology, Bern University Hospital and University of Bern, 3010 Bern, Switzerland; 5 Unit of Functional Neurosurgery, Department of Clinical and Movement Neurosciences, UCL Institute of Neurology, London WC1B 5EH, UK; 6 Wellcome Trust Centre for Neuroimaging, UCL Institute of Neurology, London WC1N 3AR, UK; 7 Department of Clinical Health, Aarhus University, DK-8200 Aarhus, Denmark; 8 Department of Neurology, Charitè—Universitätsmedizin Berlin, 10117 Berlin, Germany

**Keywords:** deep brain recording, Parkinson’s disease, hidden Markov modelling, time-series analysis, machine learning

## Abstract

Exaggerated local field potential bursts of activity at frequencies in the low beta band are a well-established phenomenon in the subthalamic nucleus of patients with Parkinson’s disease. However, such activity is only moderately correlated with motor impairment. Here we test the hypothesis that beta bursts are just one of several dynamic states in the subthalamic nucleus local field potential in Parkinson’s disease, and that together these different states predict motor impairment with high fidelity.

Local field potentials were recorded in 32 patients (64 hemispheres) undergoing deep brain stimulation surgery targeting the subthalamic nucleus. Recordings were performed following overnight withdrawal of anti-parkinsonian medication, and after administration of levodopa. Local field potentials were analysed using hidden Markov modelling to identify transient spectral states with frequencies under 40Hz.

Findings in the low beta frequency band were similar to those previously reported; levodopa reduced occurrence rate and duration of low beta states, and the greater the reductions, the greater the improvement in motor impairment. However, additional local field potential states were distinguished in the theta, alpha and high beta bands, and these behaved in an opposite manner. They were increased in occurrence rate and duration by levodopa, and the greater the increases, the greater the improvement in motor impairment. In addition, levodopa favoured the transition of low beta states to other spectral states. When all local field potential states and corresponding features were considered in a multivariate model it was possible to predict 50% of the variance in patients’ hemibody impairment OFF medication, and in the change in hemibody impairment following levodopa. This only improved slightly if signal amplitude or gamma band features were also included in the multivariate model. In addition, it compares with a prediction of only 16% of the variance when using beta bursts alone.

We conclude that multiple spectral states in the subthalamic nucleus local field potential have a bearing on motor impairment, and that levodopa-induced shifts in the balance between these states can predict clinical change with high fidelity. This is important in suggesting that some states might be upregulated to improve parkinsonism and in suggesting how local field potential feedback can be made more informative in closed-loop deep brain stimulation systems.

## Abbreviations

DBS: deep brain stimulation
HMM: hidden Markov modelling
LFP: local field potential
STN: subthalamic nucleus
TDE-HMM: time delay embedded hidden Markov model
UPDRS: Unified Parkinson’s Disease Rating Scale

## Introduction

Parkinson’s disease is a neurodegenerative condition involving degeneration of dopaminergic neurons in the substantia nigra of the midbrain and characterized by the cardinal symptoms of bradykinesia, rigidity and tremor. Symptomatology is partially reversed by the dopamine prodrug levodopa, but more advanced disease may necessitate deep brain stimulation (DBS). This therapy involves the chronic electrical stimulation of key targets in the basal ganglia, like the subthalamic nucleus (STN), that have been functionally compromised by the effects of dopaminergic denervation.^1^ The effectiveness of DBS has increased interest in the nature of activity in the neural circuits modulated by stimulation. Pathological synchronization in the beta (~20 Hz) frequency band has emerged as a key abnormality in Parkinson’s disease linked to motor impairment. ^2^ In particular, the reduction in mean beta power in the local field potential (LFP) recorded in the STN after administration of levodopa or during DBS is positively correlated with the attendant improvement of motor impairment.^3–11^

More recently, it has been stressed that beta activity consists of transient bursts in Parkinson’s disease and that burst characteristics such as rate and duration may correlate more strongly with motor impairment than mean beta power.^12–15^ The link between STN beta power, and particularly beta bursts, and motor impairment has prompted consideration of this signal as a feedback marker suitable for the closed-loop control of DBS. Closed-loop control may potentially be at least as effective as conventional, continuous DBS, and in acute studies incurs fewer stimulation-induced side effects, such as speech impairment and dyskinesias.^16–21^

However, in the correlative studies cited above, beta power, or even beta bursting, is only moderately indicative of the severity of motor impairment. So what of other oscillatory activities in the STN LFP? Broadly, the STN LFP can be divided into lower-frequency activities in the theta, alpha, low beta and high beta bands and higher frequency activities such as finely tuned gamma^22–24^ and high-frequency oscillations.^7,9,25^ Higher-frequency activities have lower amplitude in the STN, and are therefore challenging to track with the ultra-low-power electronics needed for chronic DBS systems (see, for example, Fig. 2 in López-Azcárate et al.^7^). Moreover, two key higher-frequency activities, the finely tuned gamma band feature and high-frequency oscillations, are also frequently absent. ^7,26,27^ Lower-frequency activities, on the other hand, are more consistent, of greater amplitude and more easily tracked by chronically implanted bidirectional DBS systems. However, although registerable, with the exception of low beta activity, their relationship to motor impairment has hitherto been obscure.

Here, we use hidden Markov modelling (HMM) to objectively detect different types of LFP states in the theta (4–7 Hz), alpha (8–12 Hz), low beta (13–21 Hz) and high beta (22–35 Hz) frequencies.^28,29^ When a given state visits lasts more than one oscillation cycle this may be considered a burst. We define how LFP states are modulated by dopaminergic therapy and how changes in state characteristics predict changes in parkinsonian motor impairment. In so doing we demonstrate that motor impairment is predicted not only by the predilection for episodes of low beta synchronization, but also by the rate of occurrence and the nature of state visits in the theta, alpha and high beta frequency bands. Together these different states compete to determine motor impairment with high fidelity in patients with Parkinson’s disease.

## Materials and methods

### Subjects and surgery

We investigated spectral states in the STN LFP before and after administration of levodopa in 32 patients (64 hemispheres) with advanced Parkinson’s disease undergoing DBS surgery targeting the STN. Patients had an average age of 58.5 years (range 34–79 years) and an average disease duration of 11.7 years (range 5–18 years). Twelve were female (Supplementary Table 1). All patients were diagnosed with Parkinson’s disease according to the Queen Square Brain Bank criteria.^30^ The mean of the part III of the Unified Parkinson’s Disease Rating Scale (UPDRS) score OFF medication was 37.9 (range 15–72) while the mean score ON medication was 14.4 (range 3–30). The DBS electrodes were model 3389 (Medtronic) with four platinum–iridium cylindrical surfaces of 1.27 mm diameter, 1.5 mm length and 2 mm centre-to-centre separation. The contacts were numbered 0 (lowermost) to 3 (uppermost). Correct placement of the DBS electrodes was confirmed by intraoperative microelectrode recordings in 18 patients and by postoperative imaging in all patients. All experimental procedures had received prior approval from the local research ethics committee in accordance with the standards set by the Declaration of Helsinki, and patients gave their written informed consent.

### Experiment and recordings

DBS electrodes were temporarily externalized prior to connection to the implantable pulse generator. LFP recordings were performed with the patient quietly seated with their eyes open following overnight withdrawal of anti-parkinsonian medication and were repeated before and after administration of levodopa (see below) 3–7 days after lead implantation. In 18 patients LFPs were recorded online from adjacent bipolar contact pairs (01, 12, 23) to limit volume conduction.^31^ These LFP signals were amplified and filtered at 1–250 Hz using a custom-made, high-impedance amplifier (which had at its front end input stage an INA 128 instrumentation amplifier, Texas Instruments) and recorded through a 1401 analogue/digital converter (Cambridge Electronic Design) onto a computer using Spike2 software (Cambridge Electronic Design). Signals were sampled at either 625, 1 or 2.4 kHz. Fourteen patients were recorded using the EEG system integrated in a CTF 275-channel magnetoencephalograhy scanner (CTF/VSM MedTech). Their signals were filtered 1–600 Hz and sampled at 2.4 kHz. Four LFP channels were recorded on each side, referenced to a cephalic reference. When LFP recordings were not recorded directly bipolarly, they were converted offline to a bipolar montage between adjacent contacts (three bipolar channels per side) to limit the effects of volume conduction.

### Clinical assessment

Patients were evaluated using the UPDRS part III after omitting all dopaminergic medication overnight and after administration of at least 200 mg of levodopa, with the exception of case 11 who received 100 mg of levodopa. Clinical evaluation was performed at the same time as LFP recordings, except for 14 patients in whom it was performed up to 3 months preoperatively. Changes in motor impairment with levodopa was calculated using hemibody scores for bradykinesia–rigidity (sum of UPDRS motor score sub-items 22–26 including finger taps, open and close hand movements, rapid alternating pronation and supination hand movements and leg movements) and tremor (sum of UPDRS motor score sub-items 20–21 for arm and leg), either in combination or separately, contralateral to the recording site. The clinical improvement was defined as the percentage of the reduced UPDRS hemibody score in ON condition compared with OFF condition.

### Signal processing and determination of bursts

**Figure 1:**
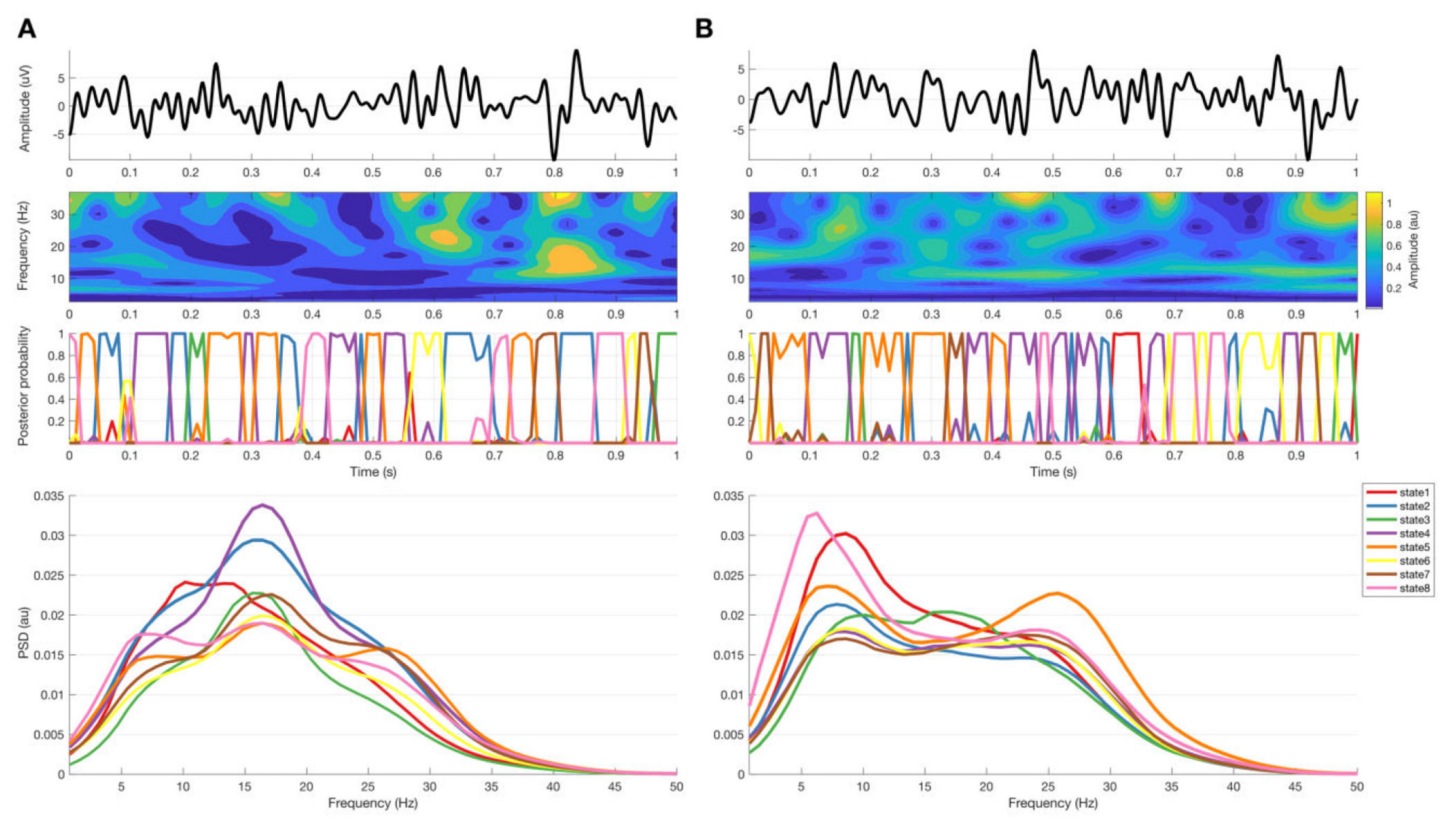
Data recorded during OFF (A) and ON (B) medications. *Top row* is raw LFP of 1-s data sample; *second row* is the corresponding amplitude of the wavelet transform; *third row* is the corresponding HMM states posterior probability (with 8 states and 11 lags). *Bottom row* are the spectra corresponding to the HMM states derived from the complete recording in all subjects.

Figure 1 and Supplementary Fig. 1 illustrate the processing steps involved in extracting various LFP states and ascribing them to the theta, alpha, low beta and high beta bands, or to a background state if no dominant peak existed. After visual signal inspection and artefact detection and rejection using Spike2 Software (CED, Cambridge, UK) and OSL [OHBA Software Library; Oxford Centre for Human Brain Activity (OHBA), University of Oxford, Oxford, UK; https://ohba-analysis.github.io/osl-docs/], data were imported into MATLAB (version R 2019 b; MathWorks, Natick, MA), where all further signal processing steps took place. Signal durations ranged from 127.9 to 639.9 s with a mean ± standard deviation (SD) signal duration of 392.4 ± 111.9 s for both the OFF and the ON conditions (data duration OFF and ON medication was matched in each subject).

The bipolar contact pair with the highest beta power in the average of the OFF and ON drug conditions was selected for further analysis based on the presumption that this contact pair was most likely to pick up from the dorsolateral ‘motor’ STN.^6,32,33^ The signals recorded in each medication condition from each hemisphere were bandpass filtered at 2–48 Hz, resampled at 100 Hz and z-normalized on a per-session basis to facilitate comparison across different recording systems and to minimize the effects of targeting variance. All processed LFPs were then concatenated into one vector representing the selected contact pair from all medication conditions and hemispheres. The concatenated signal was then fed to the time-delay embedded hidden Markov model (TDE-HMM), which was compared against a percentile thresholding model.

### Time-delay embedded hidden Markov model for burst detection

A principled way to detect bursts is to use HMMs. This can overcome many limitations of standard threshold-based approaches to burst detection, such as an overdependence on the choice of arbitrary thresholds.^28^ The approach used here, i.e. TDE-HMM, is also able to concurrently detect different types of bursts, each with distinct spectral content, without the need to predefine frequency bands of interest.

In general, HMMs assume that a time series can be described using a hidden sequence of a finite number of states.^28,29,34^ This assumes that at each time point only one state is active (‘visited’); although, as probabilistic Bayesian inference is used here, a probability of being active is assigned to each state at each time point. The HMM then assumes that the data observed in each state are drawn from a probabilistic observation model, i.e. a probability distribution, where the distribution parameters are different for each state.

The variant of HMM used here is the TDE-HMM. This uses a multivariate Gaussian distribution as the observation model, but, crucially, the HMM is inferred on a TDE version of the (z-normalized and concatenated) LFP data; i.e. copies of the LFP data, each shifted by different time lags which define the time window around the point of interest, are fed in concurrently as multiple data channels to the HMM inference. Each HMM state then captures periods of time that have distinct autocovariance, which translates into distinct spectral content. The net result is that the TDE-HMM models the data as a sequence of state visits, where each state can be interpreted as a transient spectral event of a certain type.^28^ Note that the TDE-HMM approach is also able to identify the frequency of signal segments that are shorter than the period of the signal frequency.^29^

### Data analysis and statistics

States were estimated using the TDE-HMM model for 56 different combinations of state number K (5–18 states) and time lags L (3–15 lags). The time lag L determines the window duration so that a lag of 10 means that the window includes 10 data-points either side of that representing the time under analysis. Longer windows allow better frequency resolution at the expense of temporal resolution (analogous to the time–frequency trade-off when choosing a basis set in wavelets). The state number K denotes the number of states to be detected. For every combination, TDE-HMM states were categorized by their predominant frequency content by cross-correlating the states’ time courses with the Hilbert envelopes of the theta, alpha, low beta and high beta frequency bands. Supplementary Fig. 1 illustrates the process of ascribing HMM states to different frequency bands. If the two most positive correlations were either within low (theta and alpha) or high frequencies (low and high beta), then the corresponding state was assigned to the band with highest correlation. Otherwise, states were assigned to ‘background’ activity. More than one HMM state could be attributed to a given frequency band. The analysis of multiple different models allows consistent features to emerge, while transformation from HMM states to frequency bands was used to facilitate comparisons across different HMM models and their interpretation, especially with respect to existing literature.

This assessment was repeated for the OFF and ON medication conditions. Various extracted measures of interest were extracted from the TDE-HMM models, and changes in them between the two medication conditions were correlated with the contralateral UPDRS hemibody scores to investigate any link to parkinsonian motor impairment. The extracted measures were: probability of transitioning between different states and within states (where a frequency band comprised several HMM states), fractional occupancy (referring to how much time the HMM spends in each state on average), life time (defined as the average duration per visit of each state), interval time (defined as the average time between each state visit) and switching rate between states. Occurrence rate (how often a state gets visited over time) and relative number of visits were deduced from the life time measure as further features describing bursting dynamics. The occurrence rate and relative number of visits are related to the burst rate and relative burst rate reported with the thresholding technique and described below. Inherently, some of these different summary measures of the bursting dynamics are interrelated, e.g. a state with an increase in occurrence or burst rate will see a corresponding decrease in interval time if the probability of transitioning out of that state remains the same. As such, some of these measures if associated with motor impairment might overall tend to correlate with motor state with opposite polarity, such as the occurrence or burst rate in a given frequency band and the interval time.

HMM method results were contrasted with those from the standard thresholding method of defining beta bursts, to allow comparison with relevant previous work.^12,13,35,36^ The one exception was that we did not apply an arbitrary minimum duration for bursts, so that results would parallel as closely as possible those of the HMM models. Different percentile amplitude thresholds (55th–99th in steps of 2 percentiles) were used. For each percentile, the same amplitude threshold was applied on both medication conditions, where that was calculated by taking the mean of amplitude thresholds for both ON and OFF conditions.^13^ Measures equivalent to those extracted from the TDE-HMM were extracted, where possible, from the results of the different standard thresholding models and the results combined (Supplementary Fig. 2). These features included bursting rate, relative number of bursts of different durations (<0.1, 0.1–0.2, …, 0.8–0.9, >0.9 s) and the average burst duration or life time of states in the theta, alpha, low beta and high beta frequency bands.

Ridge regression was used to create parsimonious regression models given that the number of extracted features (i.e. regressors) could exceed the number of STN recordings.^37–40^ Regression was performed on every single HMM model separately using the full set of features extracted before collapsing states into five bands (theta, alpha, low beta, high beta and background). Ridge regression uses the equation below to estimate ridge coefficients, $\hat{\beta }$ :

(1) $$\hat{\beta }={\left({\text{X}}^{\text{T}}\text{X}+\kappa \text{I}\right)}^{-1}{\text{X}}^{\text{T}}y$$

where X is the features matrix, y is the predicted score, k is the ridge parameter and I is the identity matrix. Small, positive values of k improve the conditioning of the problem and reduce the variance of the estimates. While biased, the reduced variance of ridge estimates often results in a smaller mean squared error when compared to least-squares estimates. The k parameter was set by varying the k-value in the model between –30 and 30, and the final k-value chosen when model prediction power converged and stopped changing. To avoid overfitting, we performed leave-one-out cross-validation. Finally, to evaluate the results we used the previously described method to ascribe states defined in each HMM model to our chosen frequency bands so that we could then attribute the corresponding ridge coefficients to these frequency bands.

Note that the TDE-HMM approach is also able to identify the frequency of signal segments that are shorter than the period of the signal frequency.^29^ As the content of such short segments is statistically indistinguishable from that of longer segments we included both short and long state visits, but distinguished between the contributions of state visits of differing duration in the results. This limited the number of additional constraints on the data (no minimum duration), and we have included the results of simple thresholding without a minimum duration constraint for direct comparison.

Statistical analyses were performed using MATLAB (2019b, Mathworks, Natick, MA, USA) and SPSS (2019, IBM Corp., Armonk, NY, USA). The HMM and thresholding extracted features were tested with MATLAB Lilliefors test to check whether they were normally distributed. State feature and other distributions were tested against the null hypothesis using permutation testing.^41^ Where several permutation tests were applied to the same datasets these were then corrected for multiple comparisons using the false discovery rate (FDR) method. Data are presented in the form of modified box-and-whisker plots with a box from the first quartile to the third quartile, a vertical line drawn through the box at the median and whiskers drawn up to the upper and lower extreme values (excluding outliers).

## Results

### Medication-induced changes in STN LFP states

TDE-HMM was run on the STN LFP data to identify a temporal sequence of states in each STN LFP channel, where each state can be interpreted as a transient spectral event of a certain type, including, but not limited to, beta bursts.^28^ Motor impairment in Parkinson’s disease is partially reversible after oral treatment with the dopamine prodrug levodopa. Accordingly, we began by determining which LFP states change following levodopa treatment before seeking correlations between these changes and the improvement in motor impairment. Fifty-six TDE-HMM models of the STN LFP with different numbers of states (5–18) and timeembedding lags (3–15) were separately tested ON and OFF medication.

Initially, we focused on activities with frequencies under 40 Hz, as these are more consistently found and more readily registered with the ultra-low-power amplifiers that are necessary in chronic recording systems that might, in the future, deliver closed-loop DBS. Identified LFP states with clear spectral peaks were then ascribed to the theta, alpha, low beta and high beta bands, or to a background state if no dominant peak existed (Supplementary Fig. 1). Overall, following the dopamine prodrug levodopa, LFP states in the low beta band became less common and relatively briefer, whereas those in the theta band and, to a lesser extent, the alpha band became more common and relatively longer in duration. Different TDE-HMM models could differ in the extent to which they identified levodopa-induced changes in LFP states and in their ability to predict change in motor impairment. Accordingly, in the following we focus on results that have been collapsed across all 56 TDE-HMM models.

**Figure 2:**
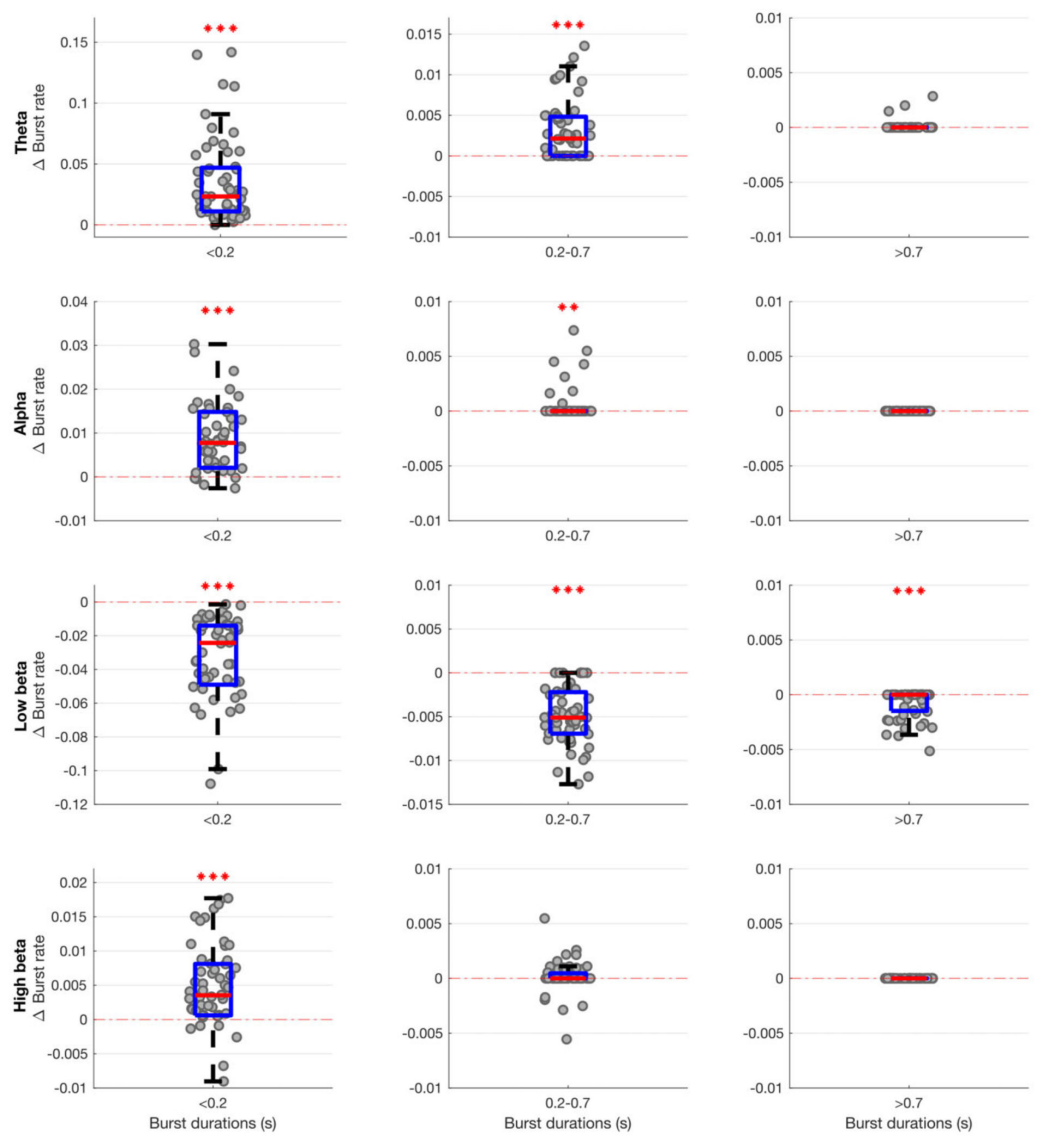
Box-and-whisker plots of change (ON–OFF) in the rate of theta (4–7 Hz), alpha (8–12 Hz), low beta (13–21 Hz) and high beta (22–35 Hz) states identified by the combination of different HMM models. There are widespread changes in the burst rate of states between the OFF and ON medication condition, with these most marked for theta and low beta states. Each dot represents the median value across the 64 hemispheres in one HMM model (data from 56 different models are plotted). Statistics were derived after performing permutation testing and thereafter corrected for multiple comparisons using the FDR method. Data are presented in the form of modified box-and-whisker plots with a box from the first quartile to the third quartile, a vertical line drawn through the box at the median and whiskers drawn up to the upper and lower extreme values (excluding outliers). *P < 0.05, **P < 0.01, ***P < 0.001.

**Figure 3:**
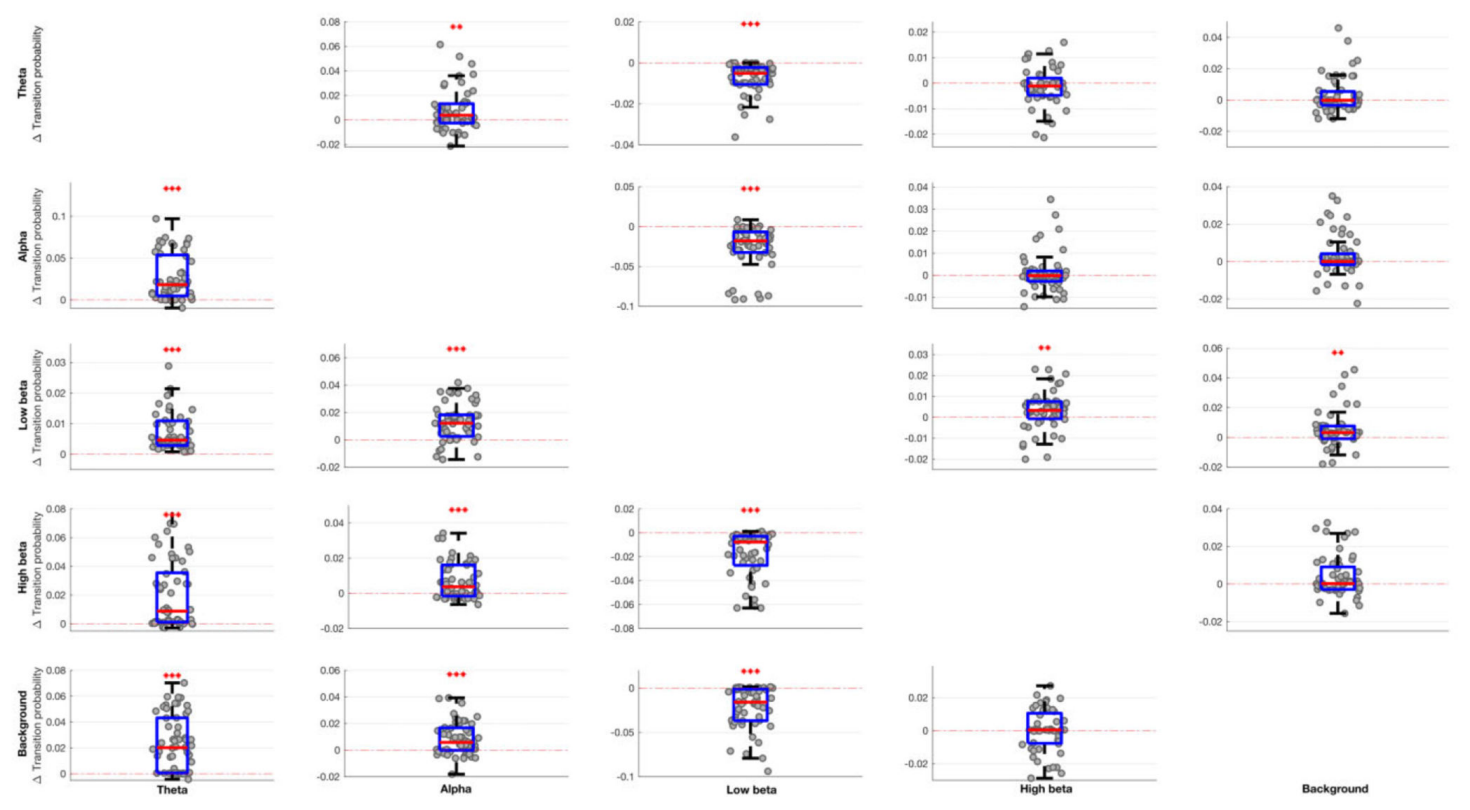
Box-and-whisker plots of medication-linked change in transition probability between various state bands (theta, alpha, low beta, high beta and background) for different HMM models. Medication significantly drives activity away from the low beta state (shown as negative change) and into the theta and alpha states (shown as positive change). Changes in the high beta and background states are less marked, with the exception that transitions of the low beta state to the high beta and background states are increased by medication. Each dot represents the median value across the 64 hemispheres in one HMM model (data from 56 different models are plotted). Statistics were derived after performing permutation testing and thereafter corrected for multiple comparisons using the FDR method. Data are presented in the form of modified box-and-whisker plots with a box from the first quartile to the third quartile, a vertical line drawn through the box at the median and whiskers drawn up to the upper and lower extreme values (excluding outliers).*P < 0.05, **P < 0.01, *** *P* < 0.001.

The occurrence rate of LFP states (or burst rate, i.e. how often a state gets visited over time) significantly increased when ON compared to OFF levodopa for the state visits of the shortest duration in all frequency bands, except low and high beta (Fig. 2). In contrast, the occurrence rate of LFP states decreased in the low beta band regardless of state duration ON compared to OFF levodopa. These changes could represent shifts from one spectral band state to another, or shifts to and from the background LFP state. This issue was addressed by considering state transition probabilities (Fig. 3). Dopaminergic medication significantly drove subthalamic activity away from the low beta state to the theta, alpha, high beta and background states, in so far as these latter states were more likely to follow the low beta state than precede it on medication. In addition, high beta and background states were significantly driven into the theta and alpha states, and the alpha state further driven into the theta state by levodopa.

To compare the present findings in the beta band with previously reported results of the standard thresholding technique, we also plotted the change in the relative rate of different durations of states (compared to the rate of all durations of states) within the beta frequency band with levodopa (Supplementary Fig. 3). There was a significant increase in the relative rate of short-duration low beta states and a decrease in the relative rate of longer duration low beta states ON levodopa. This is consistent with previous reports using the thresholding technique.^13,15^

**Figure 4:**
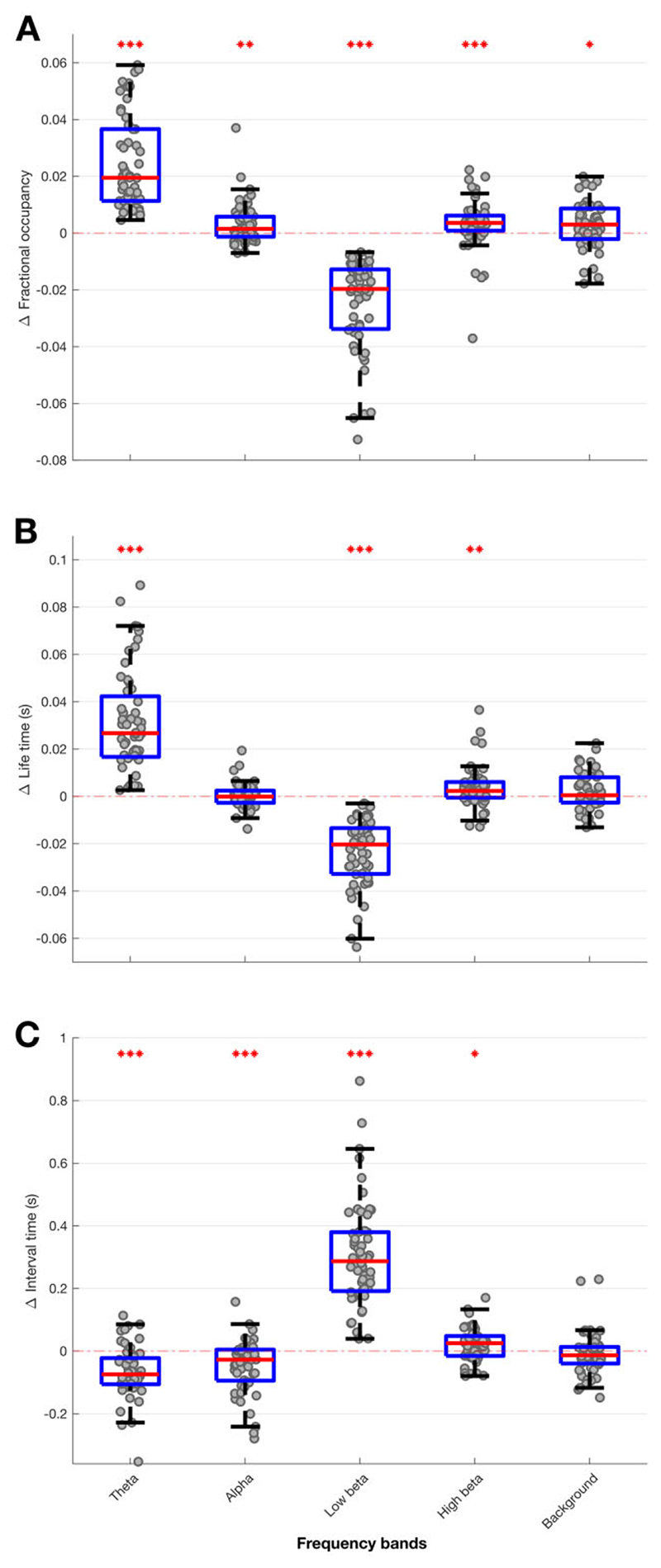
Box-and-whisker plots of medication-induced change (ON–OFF medication) in fractional occupancy (A), life time (B) and interval time (C) for different HMM models. Low beta state is reduced ON medication for fractional occupancy and life time, and increased ON medication for interval time. Theta, alpha and high beta states show the converse pattern. Each dot represents the median value across the 64 hemispheres in one HMM model (data from 56 different models are plotted). Statistics were derived after performing permutation testing and thereafter corrected for multiple comparisons using the FDR method. Data are presented in the form of modified box-and-whisker plots with a box from the first quartile to the third quartile, a vertical line drawn through the box at the median and whiskers drawn up to the upper and lower extreme values (excluding outliers). *P < 0.05, **P < 0.01, ***P < 0.001.

In addition, there were significant decreases in fractional occupancy and life time and corresponding increases in state-interval time in the low beta band ON levodopa, and significant converse changes in the theta and, to a lesser extent, alpha and high beta bands (Fig. 4). Changes in the background activity were small.

### Medication-induced changes in STN LFP states correlate with motor impairment

**Figure 5:**
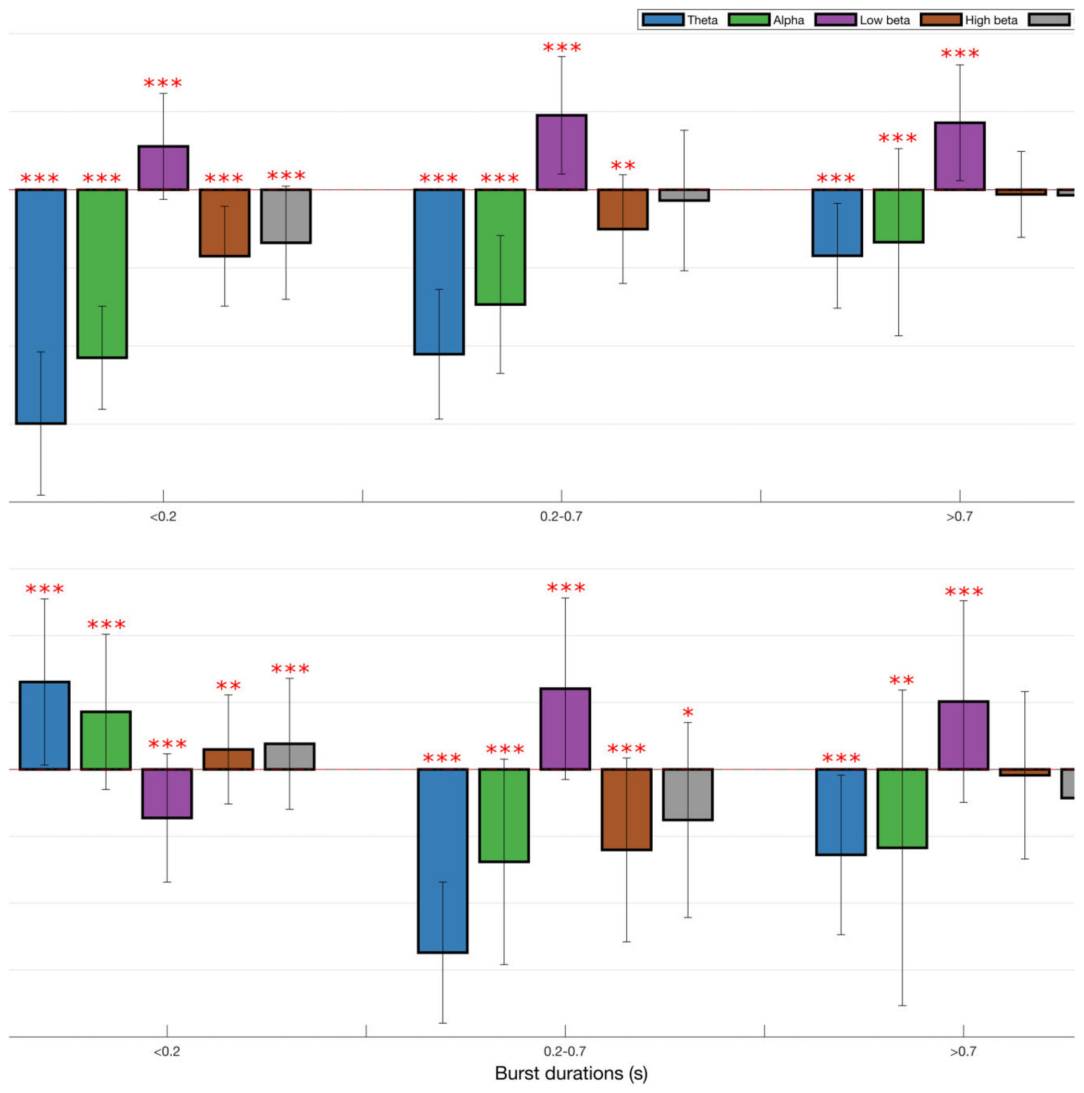
Correlation between medication-induced change (ON–OFF medication) in bursting and per cent change (ON–OFF medication) in contralateral hemibody UPDRS score including tremor items. Correlations for change in rate of states (**A**) and relative burst number (**B**) are shown for theta, alpha, low beta, high beta and background states identified by 56 different HMM models. Increase in rate of theta and alpha states is coupled with contralateral hemibody UPDRS Part III score reductions ON medication across all state durations. However, this pattern is reversed for the relative number of the shortest alpha states. Increase in rate of the low beta state positively correlates with contralateral hemibody motor UPDRS Part III score reductions ON medication across all burst durations. However, this is only true of longer durations when it comes to change in relative state numbers in the low beta band. Results were corrected for multiple comparisons using FDR method. Median correlations ± SD are shown. *P < 0.05, **P < 0.01, ***P < 0.001 and refer to whether the 56 bivariate correlations for each frequency band and state episode duration were significantly more positive or negative than zero following FDR correction.

The levodopa-induced changes in low beta and theta-alpha states were also associated with opposing effects on motor impairment (Fig. 5A). The change in the occurrence rate of the low beta states between ON versus OFF levodopa was significantly consistently positively correlated with the change in motor impairment with levodopa, so that decreases in the occurrence of low beta states were linked to improvements in motor impairment ON drug. Conversely, changes in the occurrence rates of theta and alpha band states were significantly consistently negatively correlated with change in motor impairment ON drug, so that increases in the frequency of theta and alpha states were linked to improvements in motor impairment ON drug. These contrasting effects were similar across states of different absolute durations, although the bivariate correlations themselves were relatively weak.

To compare the present findings with previously reported results of standard thresholding in the beta band, we also estimated the correlation with the relative number of states in each frequency band (Fig. 5B). As with the previous results, there was a reversal in correlation sign whereby the change in the relative occurrence rate of short duration low beta states negatively correlated with change in motor impairment and change in the relative occurrence rate of longer duration low beta states positively correlated with change in motor impairment, although the variance between different models was relatively large. Thus a relative increase in the occurrence rate of short duration beta states was linked to diminution in motor impairment ON medication, whereas a relative decrease in longer beta states was linked to less motor impairment ON drug. This is consistent with previous reports using the thresholding technique.^13^ The opposite pattern was seen in the theta state. A relative increase in the occurrence rate of short duration theta states was linked to greater motor impairment ON medication, but a relative increase in longer theta states was linked to less motor impairment ON drug. Smaller, less-consistent effects were seen in the relative number of alpha states.

### Medication-induced changes across STN LFP states predict motor impairment

**Figure 6:**
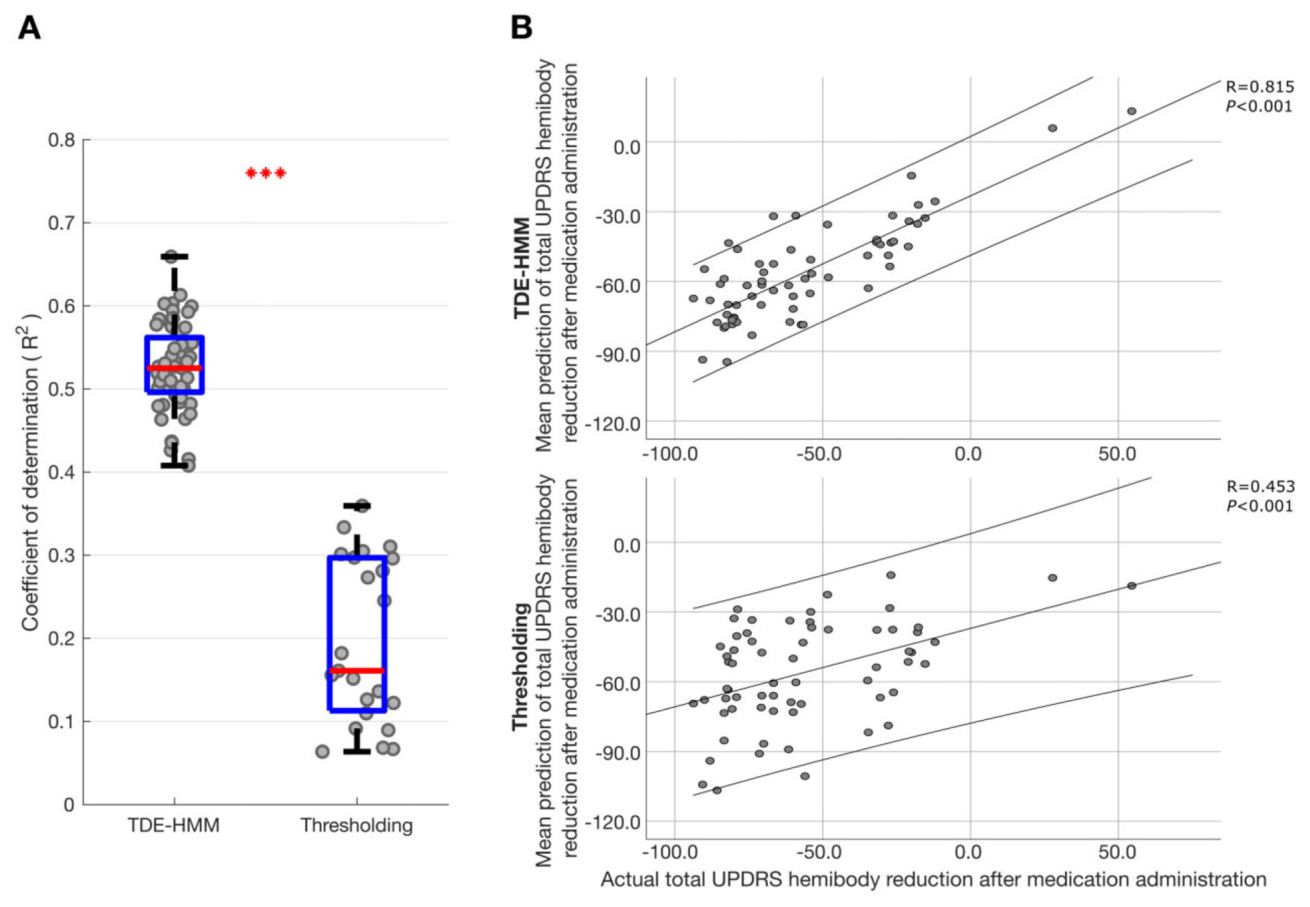
LFP HMM state prediction of change in contralateral hemibody UPDRS score including tremor items upon treatment with levodopa. (**A**) Box-and-whisker plot of coefficient of determination (*r* ^2^) between predicted and actual percentage improvement in contralateral hemibody UPDRS score including tremor items. Prediction was performed using ridge regression which utilized features extracted from different HMM models (*left*) and different percentile thresholding models across different frequency bands (*right*). Each dot represents data from one HMM model (*n* = 56) on the left and one thresholding model (*n* = 23; different threshold levels 55–99) on the right. ***P < 0.001. (**B**) Illustrative examples showing correlation between the mean predicted (across the 56 HMM and 23 thresholding models) and actual percentage improvement in contralateral hemibody bradykinesia–rigidity items of the UPDRS Part III. Prediction was performed using ridge regression which utilized multiple HMM state features (*top*) or multiple thresholded frequency-band features (*bottom*). Each dot in **B** represents data from one hemisphere in one subject (*n* = 64). Linear fits and 95% confidence limits are shown. Negative changes represent % reductions in UPDRS score after medication administration.

Changes in the absolute and relative occurrence rate of states in the different frequency bands were linked to frequency-specific and often opposing changes in motor impairment. However, the bivariate correlations reported above and illustrated in Fig. 5 are relatively modest in strength, and the statistics only confirm the consistency of the sign of frequency-specific correlations using features from different HMM models. The potential importance of the correlations lies not only in the mechanistic insight afforded, but also in the potential for the different states and their features to provide comprehensive feedback about motor impairment for closed-loop control of DBS. The question therefore arises whether multivariate models that include multiple features derived from multiple TDE-HMM states can lead to stronger predictions of motor impairment. To answer this we performed ridge regression. The regression model derived from the TDE-HMM analysis was further contrasted with that derived from standard thresholding (Fig. 6), using the same feature sets where possible. The median coefficient of determination across the 56 different TDE-HMM models was 0.52 (using leave-one-out cross-validation), indicating that the multiple regression models explained just over 50% of the variance in the change in motor impairment following medication. This is a substantial effect and contrasts with the results across multiple thresholding models using the same data, with different threshold percentiles in the theta, alpha, low beta and high beta frequency bands (23 combinations; 55th–99th percentiles). Here the median coefficient of determination was only 0.16.

We also considered the contributions made to the regression model by states in the different frequency bands and their features (Supplementary Fig. 4). The normalized regression coefficients for features in the low beta state and for those in the remaining spectral states tended to have opposing signs, in line with the contrasting effect of levodopa on these features and with the results of bivariate correlations between changes in these features and change in motor impairment with medication.

The change in contralateral UPDRS Part III hemibody scores predicted above in Fig. 6 included both the change in bradykinesia–rigidity and tremor items. Results were similar if changes in tremor items were excluded (Supplementary Fig. 5). Conversely, prediction of change in contralateral hemibody tremor scores alone was weaker than prediction of change in bradykinesia–rigidity, although the narrower range of changes in tremor score may have contributed to this difference (Supplementary Fig. 6). Still, ridge regression based on TDE-HMM features was able to predict about 30% of the variance in the change in contralateral tremor scores. This was greater than the prediction of variance in the change in contralateral tremor scores with thresholding models (Supplementary Fig. 6). It was also greater than the prediction of tremor change using just bradykinesia–rigidity scores, which was 8%, suggesting that some LFP features were of additional benefit in predicting tremor variance. We also considered the contributions made to the regression model for tremor by states in the different frequency bands and their features (Supplementary Fig. 7).

### Both low beta and other spectral states contribute to prediction of change in motor impairment with levodopa

**Figure 7:**
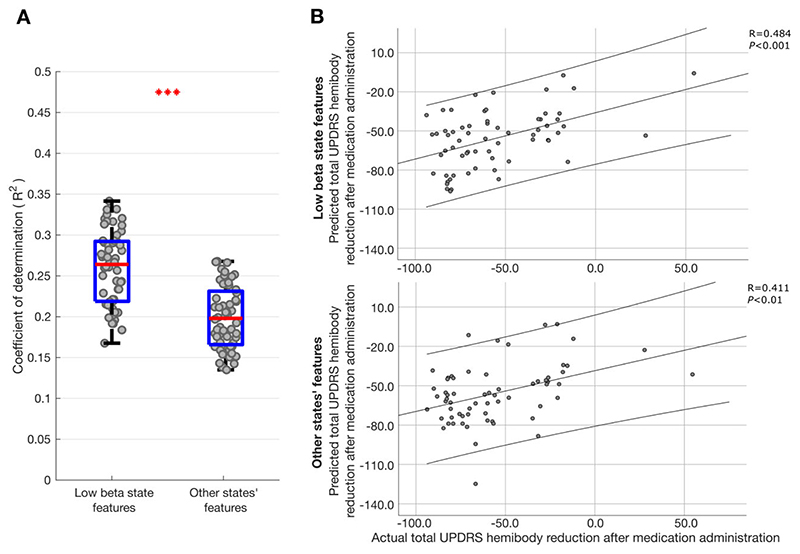
LFP HMM state prediction of change in contralateral hemibody UPDRS score upon treatment with levodopa using different feature sets. (**A**) Box-and-whisker plots of coefficients of determination (*r* ^2^) between predicted and actual percentage improvement in contralateral hemibody UPDRS score including tremor items, estimated by ridge regression utilizing only low beta features (*left*) and theta, alpha and high beta features (*right*) extracted from different HMM models. Each dot represents data from one HMM model (n = 56). *** *P* < 0.001. (**B**) Illustrative example of single HMM model showing correlation between predicted and actual percentage improvement in contralateral hemibody UPDRS score including tremor items. Prediction was performed using ridge regression which utilized only low beta (*top*) or utilized theta, alpha and high beta features (*bottom*) from an 8-state HMM model. Results of leave-one-out cross-validation are presented for the two ridge regression models. Each dot in **B** represents data from one hemisphere in one subject (*n* = 64). Linear fits and 95% confidence limits are shown. Negative changes represent per cent reductions in UPDRS score after medication administration.

The normalized ridge regression coefficients reported in Supplementary Fig. 4 suggested that STN LFP states at frequencies other than low beta also contributed to the prediction of change in motor impairment with levodopa, albeit their effect was opposite in sign to that of the corresponding features in the low beta state. Ridge regression creates parsimonious regression models that generalize well when doing prediction, even in the presence of collinearity between multiple regressors (such as the occurrence rate and relative burst rate which themselves were deduced from the life time measure).^36^ However, in doing so the estimates of the normalized regression coefficients of the different inputs may be biased, even though they have a smaller variance than with ordinary least-squares estimators. Estimates of the normalized regression coefficients should therefore be interpreted cautiously. With this in mind we sought further evidence that low beta and other frequency states both contributed to the prediction of change in contralateral hemibody UPDRS score upon treatment with levodopa. To this end we separated those features relating to the low beta state and those relating to the theta, alpha, high beta and background states, and performed ridge regression as before on these feature subsets. Figure 7 indicates that both were able to predict the change in motor impairment with levodopa, although the respective coefficients of determination (*r* ^2^) values were about half of those of the combined model (Fig. 6A).

**Figure 8:**
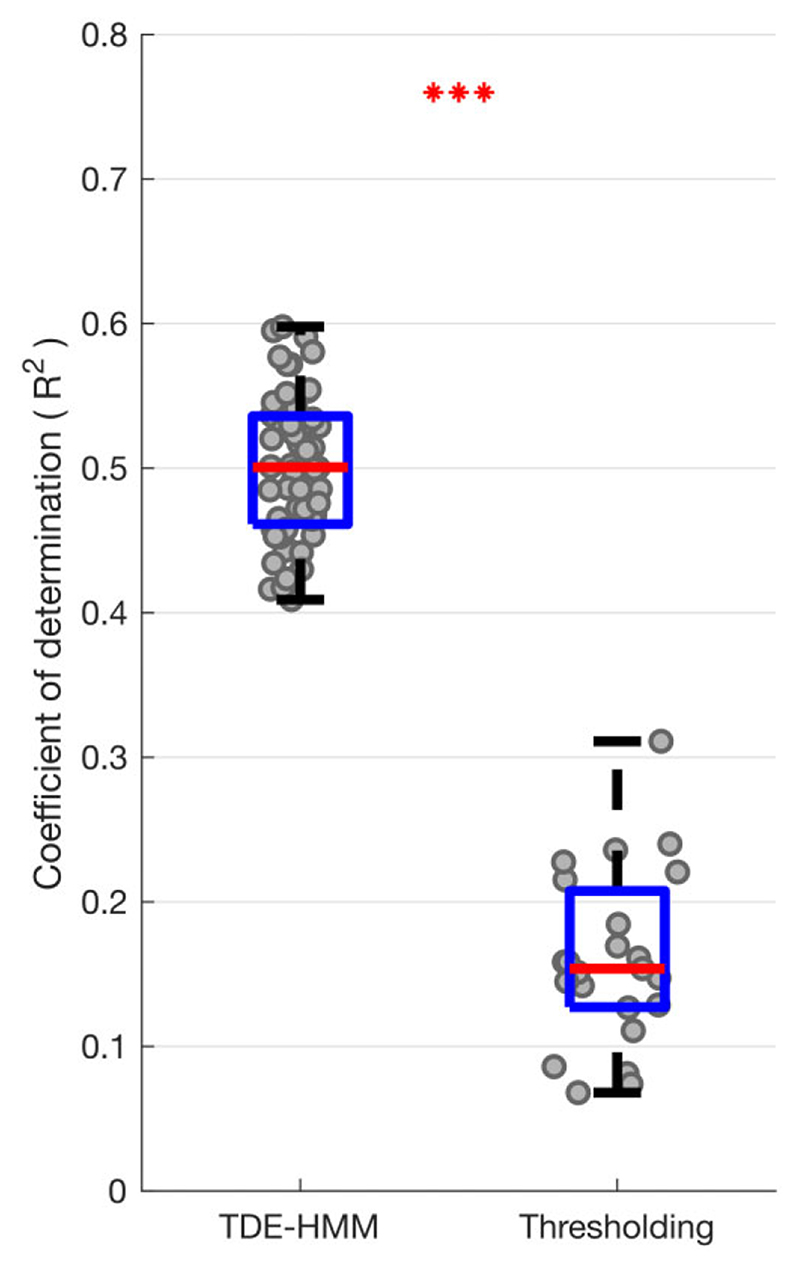
Box-and-whisker plot of coefficient of determination (*r* ^2^) between predicted and actual contralateral hemibody UPDRS score, including tremor items, OFF medication. Prediction was performed using ridge regression which utilized features extracted from different HMM models (*left*) and different percentile thresholding models based on LFPs recorded OFF medication (*right*). Each dot represents data from one HMM model (*n* = 56) on the left and one thresholding model (*n* = 23; one for each threshold applied across the four frequency bands) on the right. ***P < 0.001.

Our core analysis considered data low-pass filtered at 45 Hz but did not include amplitude information features or gamma activity. Supplementary Fig. 8 gives the results of including amplitude features in the ridge regression models illustrated in Fig. 6. This addition increased the variance in change in motor impairment with levodopa explained by TDE-HMM models to 0.57 ± 0.06 (Supplementary Fig. 8A, left) and by thresholding models to 0.22 ± 0.12 (Supplementary Fig. 8A, right). To explore the possible additional contributions of gamma activity in predicting change in motor impairment with levodopa, we performed an additional TDE-HMM analysis of the same data now pass-band filtered over 55–95 Hz. This time we identified the states with time courses that best cross-correlated with the Hilbert envelope of gamma power, where this was defined as 60–90 Hz. The gamma band state features were then added to the ridge regression models illustrated in Fig. 8A. Adding gamma features increased variance explained by TDE-HMM models to 0.59 ± 0.06 (Supplementary Fig. 8B, left) and by thresholding models to 0.23 ± 0.12 (Supplementary Fig. 8B, right). The signs of the amplitude coefficients in the ridge regression model depended on the frequency band. A reduction in amplitude in the low beta band on medication was associated with a greater improvement in motor impairment on medication, whereas the opposite was seen in the remaining frequency bands. The signs of the gamma coefficients in the ridge regression were opposite to those of corresponding low beta coefficients and similar in sign to those of the theta, alpha and high beta frequencies coefficients.

### Multivariate correlations between STN LFP states and motor impairment OFF medication

Next, we considered whether multivariate models that include multiple TDE-HMM states and their features based on LFPs recorded OFF dopaminergic medication were also able to give comprehensive prediction of the motor impairment across patients OFF medication (Fig. 8). Again, ridge regression based on TDE-HMM features performed very well, predicting about 50% of the variance in contralateral combined bradykinesia–rigidity and tremor scores OFF medication (Fig. 8), or contralateral bradykinesia–rigidity alone (Supplementary Fig. 9A), much better than ridge regression based on thresholding models. The HMM states and features contributing to the regression models predicting contralateral UPDRS Part III hemibody scores OFF medication were similar to those contributing to regression models predicting the change in contralateral UPDRS Part III hemibody scores with medication (cf. Supplementary Fig. 10 with Supplementary Fig. 4). To highlight this Supplementary Fig. 11 presents a scatter plot of all the median normalized regression coefficients from the two sets of models. These tend to lie on a diagonal, which suggests that the LFP features that predict motor impairment OFF medication are those that may be modulated by treatment with levodopa to achieve improvement in motor state.

As before, prediction of contralateral hemibody tremor scores alone was weaker than prediction of bradykinesia–rigidity, but ridge regression based on TDE-HMM features was still able to predict about 30% of the variance in the contralateral tremor score OFF medication (Supplementary Fig. 9B).

To explore the effect of expanding the feature set for prediction of OFF medication contralateral UPDRS Part III hemibody scores, we included amplitude and gamma features in the ridge regression model. The result for adding amplitude information is illustrated in Supplementary Fig. 12, which demonstrates that the variance in contralateral combined bradykinesia–rigidity and tremor scores OFF medication explained by TDE-HMM models increased to 0.54 ± 0.05 (Supplementary Fig. 12A, left) and that explained by thresholding models increased to 0.18 ± 0.07 (Supplementary Fig. 12A, right). Adding gamma features to the model illustrated in Supplementary Fig. 12A increased the variance explained by TDE-HMM models to 0.56 ± 0.06 (Supplementary Fig. 12B, left) and that explained by thresholding models to 0.18 ± 0.07 (Supplementary Fig. 12B, right). The signs of the amplitude coefficients depended on the frequency band as before and the signs of the gamma coefficients in the ridge regression were opposite to those of corresponding low beta coefficients.

## Discussion

We have demonstrated that multiple spectral states in the STN LFP have a bearing on motor impairment in patients with Parkinson’s disease, and that levodopa modulates these states and shifts the balance between them in a way that predicts clinical change with high fidelity. Using group-level TDE-HMM we showed that the preponderance, rate of occurrence and duration of LFP states in the low beta frequency range positively correlates with motor impairment in patients withdrawn from their anti-parkinsonian medication. Levodopa reduced the preponderance (fractional occupancy), rate of occurrence and duration of low beta states, with this reduction correlating with the improvement in motor impairment. These observations are consistent with earlier reports using a standard thresholding approach to state or burst identification in patients with Parkinson’s disease, although these did not distinguish between the low and high beta band and only determined the relative numbers of states of a given duration, and hence could not unequivocally detect whether short or long duration beta bursts were independently detrimental as measured by motor UPDRS score.^12,13^ The deleterious effects of the LFP states we identified were limited to the low beta band, in line with reports of correlations between averaged LFP power and motor impairment that are similarly limited to the low beta band,^10,42^ and studies that suggest that low- and high-frequency beta activities may be functionally distinct.^32,43,44^

In addition, bivariate correlations and the normalized regression coefficients from ridge regression models suggested that STN LFP states at other frequencies (up to 40 Hz) had opposing effects to the low beta state. Thus the preponderance, occurrence rate and duration of LFP states in the theta, alpha and high beta frequency ranges negatively correlated with motor impairment in patients withdrawn from their anti-parkinsonian medication, and levodopa increased the preponderance, occurrence rate and duration of these states, with the increase correlating with the improvement in motor impairment. Changes in LFP state behaviour were biggest in the theta frequency band. Although averaged power spectra of LFP need not faithfully mirror LFP state behaviour in the STN, there are past reports of theta and, to a lesser extent, alpha activity being increased by levodopa in Parkinson’s disease.^22,43,45–48^ These are consistent with our observation that the occurrence rates, fractional occupancies and life times of theta and alpha states were significantly increased when ON compared to OFF levodopa for the state visits of the shortest duration. Moreover, a recent study also suggested that theta increases might oppose the effects of beta bursts, as theta increased as beta bursts became disorganized and attenuated.^49^

The opposing effects of the low beta state and theta, alpha and high beta states were together able to account for just over 50% of the variance in patients’ hemibody impairment OFF medication, and in the change in hemibody impairment following ingestion of the dopamine prodrug, levodopa. The features of the different states contributing to the regression model for hemibody UPDRS scores OFF medication were similar to those contributing to the regression model predicting the change in contralateral hemibody scores with medication. This suggests that the LFP features that predict motor impairment OFF medication are those that are modulated by treatment with levodopa to achieve improvement in motor state. Hence these features, like the elements of the motor impairment which they predict, are modulated by the current status of dopaminergic activity.

Our core analysis sought features of HMM states with a frequency of under 40 Hz that predicted motor impairment. However, we did not include amplitude information and omitted the effects of gamma band activities. In a secondary analysis we therefore included these features in our ridge regression. Information on signal amplitude and inclusion of HMM states in the gamma band led to modest further increases in the prediction of motor impairment off medication and in the change in this with levodopa treatment. Perhaps the most parsimonious explanation for the modest effect of incorporating amplitude information is that the pattern of spectral states under 40 Hz determines signal amplitude or vice versa. Thus adding features related to absolute amplitude did not greatly improve predictions. Gamma activity may have had modest impact because it is not a consistent feature in LFP recordings across patients, and because the information contained within may be shared by reciprocal changes at lower frequencies.^26,27^

### Experimental considerations

The HMM approach serves to define states on objective statistical grounds and does not necessitate the application of arbitrary filtering within a canonical frequency band or of arbitrary thresholds. Indeed, the derived features were consistently more predictive of motor impairment than features drawn from simple, standard thresholding used for burst detection. However, the HMM will identify as many states as are defined in the model, and if one or more related states react in a particular way then the converse may be reflected in the remaining state features, where these are considered relative to other states. Yet this is unlikely to explain the reciprocal nature of low beta and non-low beta features, both in terms of their response to levodopa and their prediction of motor impairment. This is because multiple (5–18) states were prescribed and systematic differences between frequency bands other than low beta were identified. Thus the major reciprocal effects occurred in the theta band and the least were seen in the background activity. Moreover, had changes in the non-low beta band been secondary to those in the low beta band, then we should not have seen additional predictive value from non-low beta band features; the ridge regression model with all features was able to predict much more of the variance in motor UPDRS than the model with just low beta band features. Finally, not all of the state features considered in this study were relative to other states. Life time, interval time and occurrence rate take absolute values, and yet were also reciprocal in nature in the low beta and non-low beta bands.

A clear limitation of the HMM is that only one state is visited at any given time. Although this state will have the most evidence and can be considered dominant at that moment in time, our approach will miss independent subsidiary states that might overlap in time, as might come from other neural generators.

By focusing on frequency bands (as a *post hoc* analysis step performed after HMM state identification and feature prediction by ridge regression), we facilitated comparison with the existing literature, but this approach meant that multiple HMM states could get combined together into a given frequency band, and we could therefore be overlooking detail available in the underlying states. For example, it is possible that different HMM states could be differentially affected by medication status (e.g. change in burst rate, interval rate, etc.) and this detail gets lost when states are combined into a single frequency band. In addition, the number of states to be classified by HMM has to be predefined, as does the number of lags utilized, although by collapsing the results across many realizations of the HMM, as here, we can identify robust features in the data. We should also note that the attribution of different HMM states to canonical frequency bands was not perfect, partly because the frequency ranges of these bands are somewhat arbitrary when applied to the basal ganglia and predominantly defined on the basis of cortical physiology.

It is tempting to assume that theta, alpha and high beta states promote normal movement, but we did not assess the presence of levodopa-induced dyskinesias in our cohort and so it remains to be seen whether any of these LFP states are linked to dyskinesias ON medication. Indeed, there is some evidence that excessive theta activity in dorsolateral motor STN might be related to dyskinesias. ^22,45,46^ Gamma oscillations in the STN LFP have also been associated with dyskinesias on levodopa.^22,46,50^ Ultimately, it must be acknowledged that the link between different LFP states in the STN and motor impairment is correlational in nature, and further studies are needed to determine whether any of these LFP states are mechanistically involved in determining motor impairment.

With respect to our clinical data it should be noted that we had a mix of motor UPDRS values determined at the time of the recording in some subjects and values determined up to 3 months preoperatively in others. This may increases the variance in our clinical measure, as clinical impairment may have been greater in those assessed preoperatively given that the postoperative stun effect would have been absent. This may have potentially led us to underestimate the regression coefficients linking burst features to motor impairment. Finally, it should be noted that bursts can be defined by means other than the original thresholding method.^12^ It is possible that these newer methods might have led to increased regression coefficients estimates in the low beta band.^15,51^ However, the use of the original threshold method allowed us to more easily contrast our findings with those in the literature.^12,13,35,36^

### Implications for therapy

The most immediate therapeutic significance of the present findings lies in the potential identification of a more informative feedback signal for closed-loop DBS than beta activity alone.^52^ When all LFP states and corresponding features were considered in a multivariate ridge regression model it was possible to predict just over 50% of the variance in patients’ hemibody impairment. This is remarkable, and potentially valuable as feedback, regardless of whether the signals have mechanistic relevance. Moreover, although the strongest prediction was seen for bradykinesia and rigidity, tremor could also be predicted. The latter is not the case when beta power or bursts are considered alone.^3,5,13,42^

However, several points need addressing before the tracking of the pattern of multiple LFP states can be used as feedback. First, it should be noted that LFP state correlations with motor impairment were shown across subjects rather than within subjects. However, recent studies relating beta bursts to trial-to-trial variations in motor performance make it possible that our findings will extrapolate to the individual.^35,36,53^ Second, until there is an online version of HMM, this technique will have to be limited to the offline identification of the most informative states and state characteristics within a given subject, so that these features can then be used to focus supervised machine learning techniques that can be applied online.

Our findings are also important in suggesting that while some LFP states might be usefully downregulated for therapeutic benefit, others could potentially be upregulated to better mimic the effect of dopaminergic therapy and improve parkinsonism. Current conventional DBS serves to suppress low beta activity in the STN, but also potentially beneficial activities in other frequency bands.^54^ The present findings therefore further motivate closed-loop DBS as this is more selective in its suppression of LFP states when it is only triggered by beta bursts,^12^ and encourage the development of phase-locked stimulation techniques that can both selectively up- and downregulate target brain states.^55–57^

## Supplementary Material

Supplementary material available at *Brain* online.

Suppl1

Suppl2

## Data Availability

The original data are available upon reasonable request to the corresponding authors.
