# Supplementary material for: Balance between competing spectral states in subthalamic nucleus is linked to motor impairment in Parkinson’s disease
Source: Brain. Author manuscript; Available in PMC 2022 Apr 6. (PMC8967096; doi:10.1093/brain/awab264)
Supplement: Suppl1 [file EMS144059-supplement-Suppl1.pdf]

Subject	Sex	Age (years)	Disease duration (years)	Total UPDRS III OFF Drugs OFF DBS	Total UPDRS III ON Drugs OFF DBS
1	M	63	10	21	7
2	F	67	18	46	27
3	F	64	6	47	25
4	M	49	7	20	11
5	F	64	11	30	8
6	F	53	12	27	18
7	F	73	16	48	10
8	M	79	7	27	13
9	M	34	8	22	3
10	F	59	13	21	6
11	F	62	12	37	17
12	M	69	18	53	30
13	F	48	8	22	4
14	M	69	11	24	18
15	M	57	17	30	18
16	M	65	14	38	29
17	F	63	5	15	11
18	M	67	16	47	26
19	M	58	13	43	25
20	M	57	17	54	14
21	M	60	15	56	10
22	M	61	9	28	5
23	M	48	11	72	16
24	M	52	12	35	10
25	M	51	9	49	21
26	F	58	14	71	18
27	F	61	7	34	4
28	M	54	15	53	19
29	F	58	10	55	16
30	M	55	15	19	5
31	M	40	10	30	9
32	M	54	8	38	9
**Mean (SD)**	**M (20), F (12)**	**58.5 (9.2)**	**11.7 (3.7)**	**37.9 (15.1)**	**14.4 (7.9)**

Supplementary Table: Clinical Summary. Where UPDRS motor scores were derived with half points these have been rounded up. SD =standard deviation. All but five subjects have been previously reported (cases 12-19 in Kuhn *et al.,*[3]; cases 1, 2, 5, 7-9, 11, and 13- 17 in Litvak *et al.,*[56]; cases 1-8 Tinkhauser *et al.,*[13]).
