# Supplementary material for: Balance between competing spectral states in subthalamic nucleus is linked to motor impairment in Parkinson’s disease
Source: Brain. Author manuscript; Available in PMC 2022 Apr 6. (PMC8967096; doi:10.1093/brain/awab264)
Supplement: Suppl2 [file EMS144059-supplement-Suppl2.pdf]

Supplementary Figures

Supplementary Figure 1: Example of HMM model with 8 states and 7 lags showing the process of assigning states to frequency bands. First, HMM state time courses in the STN LFP of a given subject are correlated with the Hilbert envelopes of the four different frequency bands (theta, alpha, low beta, and high beta) in that subject. Afterwards, if the two most positive correlations were either within the 2 low frequency bands (theta & alpha) or high frequency bands (low & high beta), then the state is assigned to the band with highest correlation; otherwise, if the strongest 2 correlations were split within the low and high frequency bands, then the state is assigned to a background band (see values of correlation coefficients in the middle panel). This results in 5 state bands. This process allows comparisons across various HMM combinations (e.g. with potentially different numbers of states). Probability distribution function panel (**bottom**) shows correlation coefficients used to ascribe states defined in each HMM model to the theta, alpha, low beta and high beta frequency bands. Y-axis shows probability distribution functions and x-axis shows the correlation coefficients extracted when classifying HMM states into corresponding frequency bands.

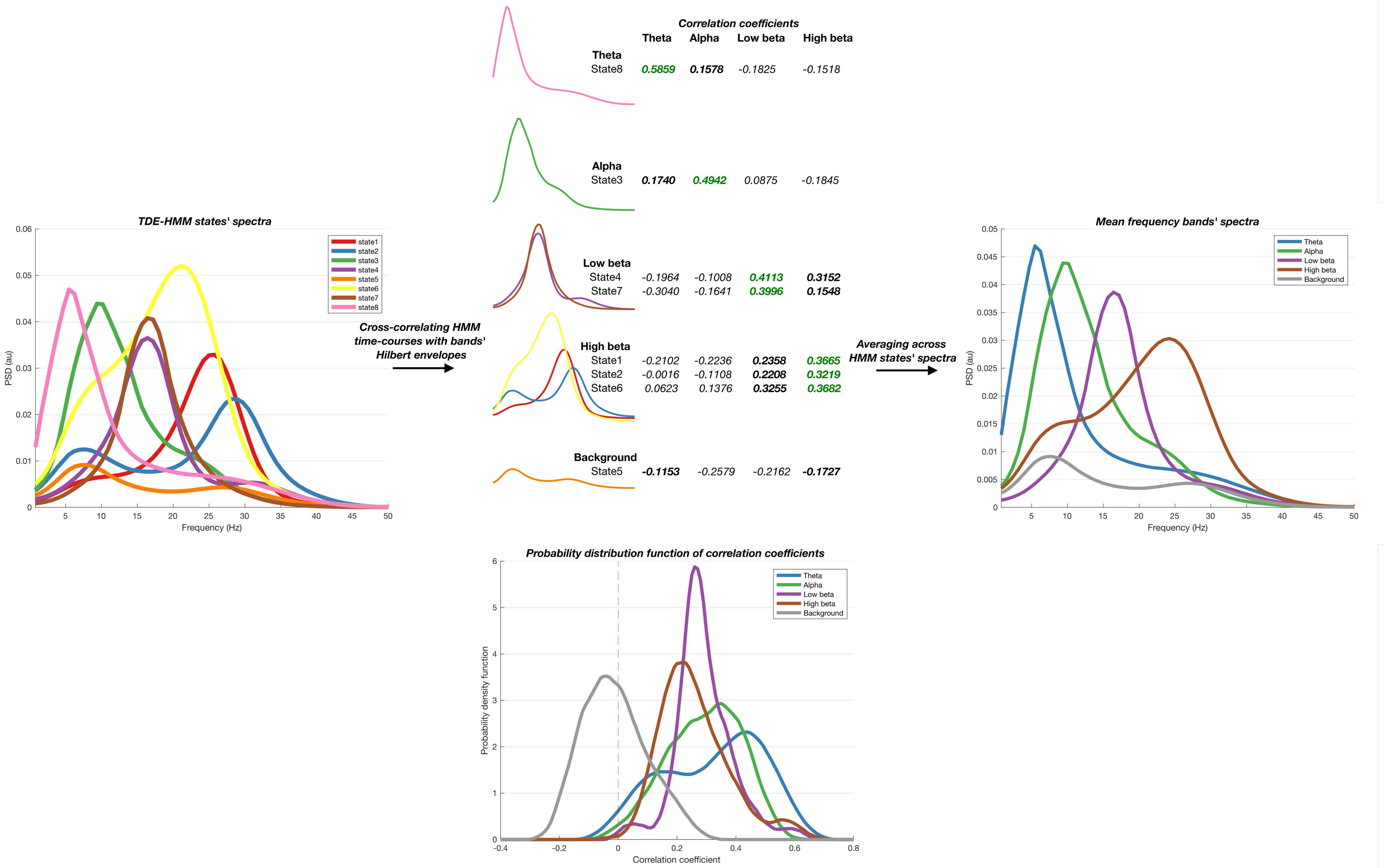
TDE-HMM states' spectra
PSD (au)
Frequency (Hz)
state1
state2
state3
state4
state5
state6
state7
state8
Cross-correlating HMM time-courses with bands' Hilbert envelopes
Correlation coefficients
Theta
Alpha
Low beta
High beta
Theta
State8 0.5859 0.1578 -0.1825 -0.1518
Alpha
State3 0.1740 0.4942 0.0875 -0.1845
Low beta
State4 -0.1964 -0.1008 0.4113 0.3152
State7 -0.3040 -0.1641 0.3996 0.1548
High beta
State1 -0.2102 -0.2236 0.2358 0.3665
State2 -0.0016 -0.1108 0.2208 0.3219
State6 0.0623 0.1376 0.3255 0.3682
Background
State5 -0.1153 -0.2579 -0.2162 -0.1727
Averaging across HMM states' spectra
Mean frequency bands' spectra
PSD (au)
Frequency (Hz)
Theta
Alpha
Low beta
High beta
Background
Probability distribution function of correlation coefficients
Probability density function
Correlation coefficient
Theta
Alpha
Low beta
High beta
Background

Supplementary Figure 2: Flow chart summarizing analysis pipeline. LFP signals are used to predict motor impairment. Features used in ridge regression methods are extracted from HMM-TDE and thresholding models. Note that burst duration refers to the rate of bursts of different duration (<0.1 s, 0.1-0.2 s, ... , 0.8-0.9 s, >0.9 s) whereas life time refers to the average burst duration of of states in the different frequency bands.

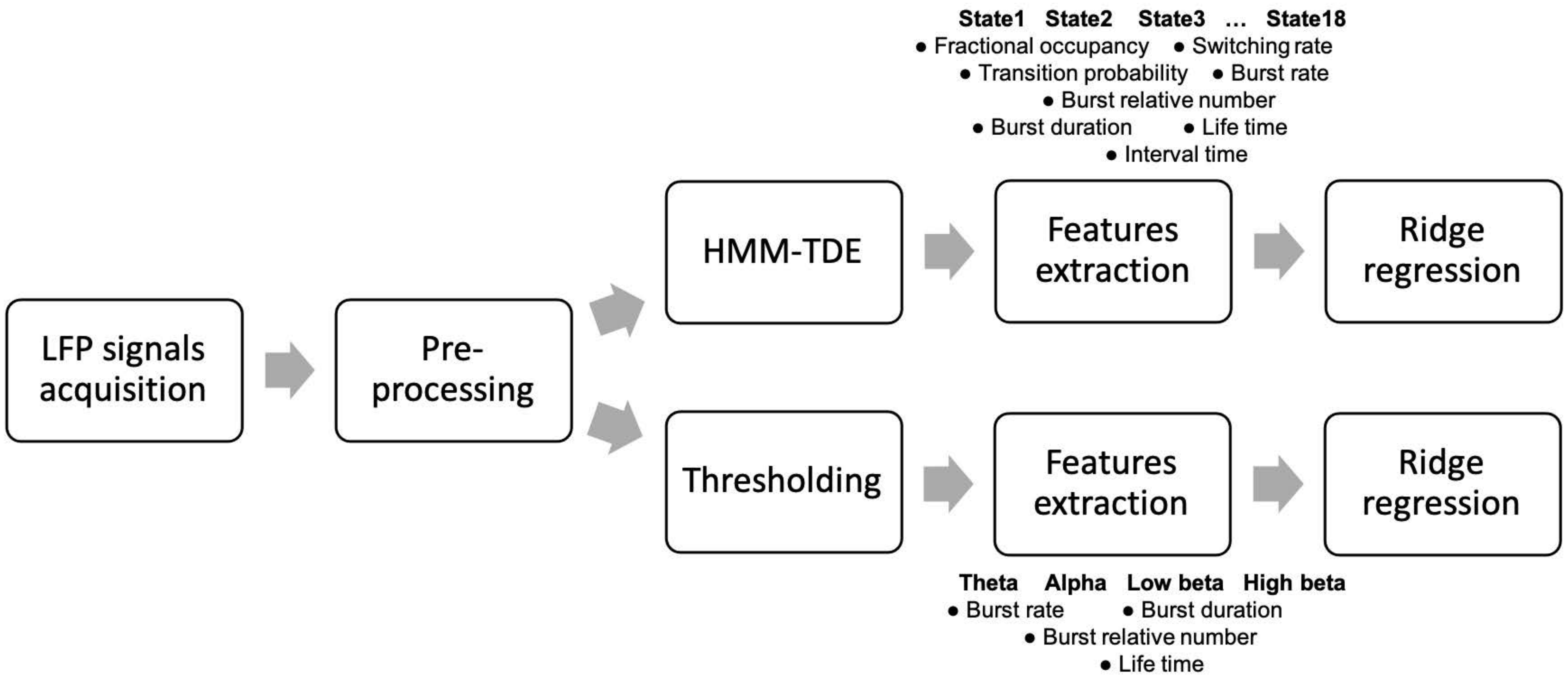
State1 State2 State3 … State18
• Fractional occupancy • Switching rate
• Transition probability • Burst rate
• Burst relative number
• Burst duration • Life time
• Interval time
LFP signals acquisition
Pre-processing
HMM-TDE
Features extraction
Ridge regression
Thresholding
Features extraction
Ridge regression
Theta Alpha Low beta High beta
• Burst rate • Burst duration
• Burst relative number
• Life time

Supplementary Figure 3: Box-and-whisker plots of change (ON-OFF) in number of theta, alpha, low beta and high beta states relative to the total number of states identified by the combination of different HMM models. There are widespread changes in the relative number of states between the OFF and ON medication condition, with these most marked in the theta and low beta bands. Each dot represents the median value across the 64 hemispheres in one HMM model (data from 56 different models is plotted). Statistics were derived after performing permutation testing and thereafter corrected for multiple comparisons using the false discovery rate (FDR) method. Data are presented in the form of modified box-and-whisker plots with a box from the first quartile to the third quartile, a vertical line drawn through the box at the median, and whiskers drawn up to the upper and lower extreme values (excluding outliers).*: $p<0.05$, **:$p<0.01$, ***: $p<0.001$.

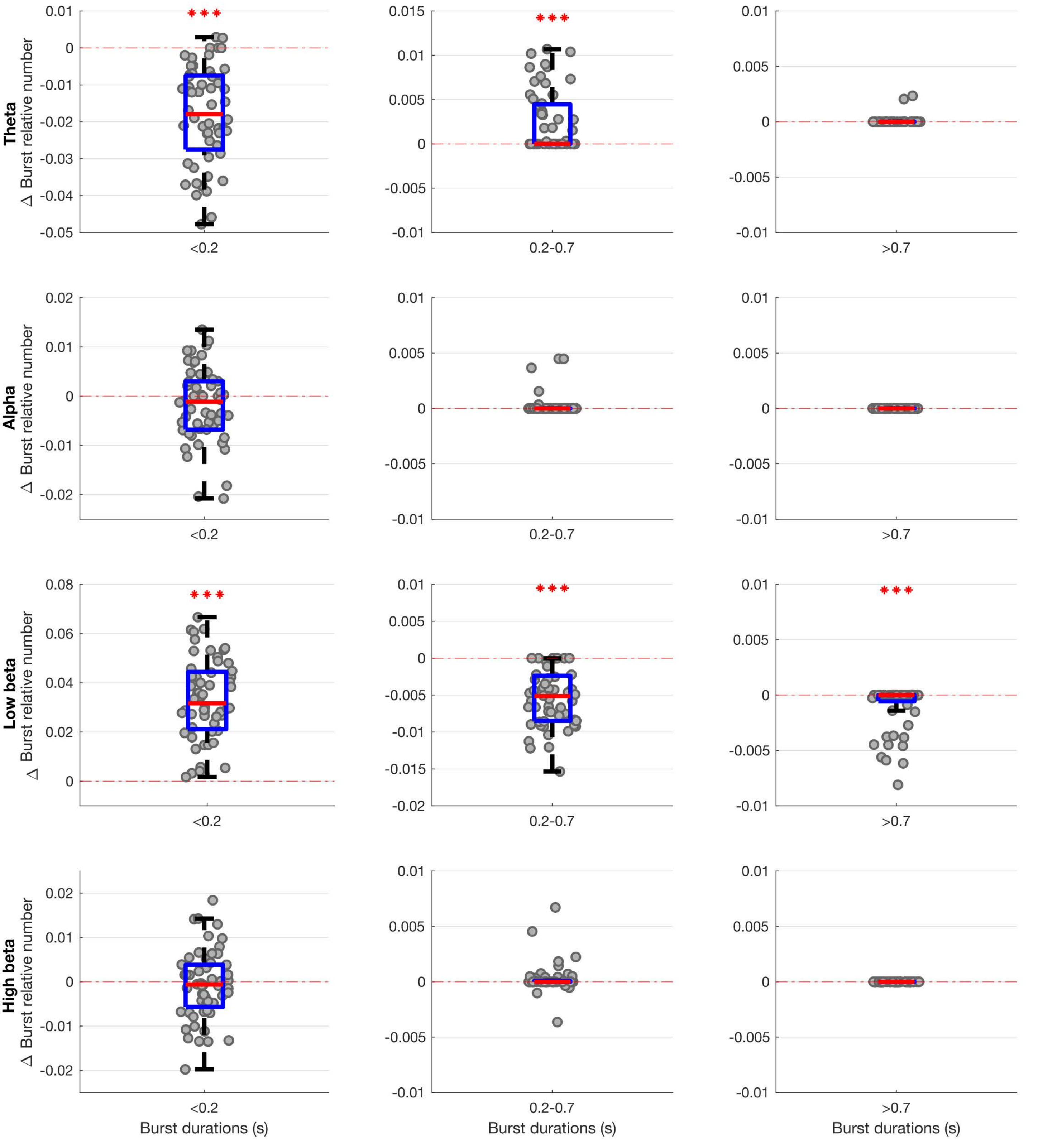

Theta
Alpha
Low beta
High beta
Δ Burst relative number
***
<0.2
0.2-0.7
>0.7
Burst durations (s)

Supplementary Figure 4: Normalised ridge regression coefficients for the features used to predict change in contralateral hemibody UPDRS score ON medication across different HMM models. Change was defined as ON-OFF hemibody UPDRS scores that included tremor items. The most negative contributions (more negative than -0.5) to the regression models came from medication-induced changes in theta; the short and long burst occurrence rate, fractional occupancy, interval time and transition probability from the theta state, although there were many other significant but weaker negative correlations. The most positive contribution (more positive than +0.5) to the regression models come from medication-induced changes in the occurrence rate of short duration low beta states, although again there were many other significant but weaker positive correlations. Median normalised regression coefficients across the 56 different HMM models ± SD are shown. *: $p<0.05$, **: $p<0.01$, ***: $p<0.001$.

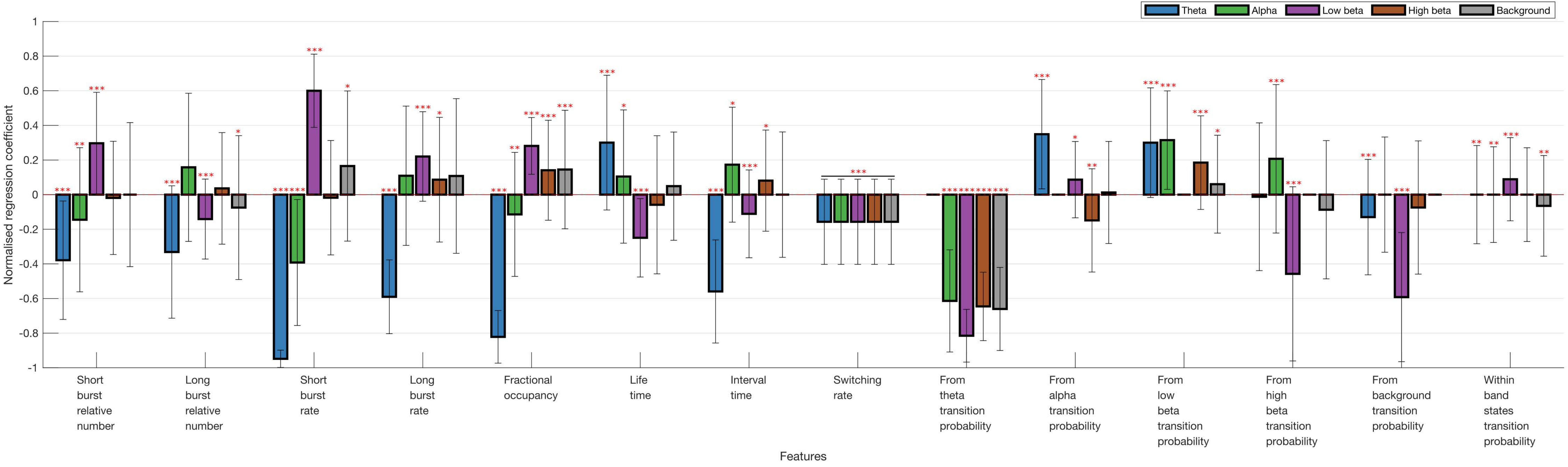

Theta
Alpha
Low beta
High beta
Background
Normalised regression coefficient
1
0.8
0.6
0.4
0.2
0
-0.2
-0.4
-0.6
-0.8
-1
Short burst relative number
Long burst relative number
Short burst rate
Long burst rate
Fractional occupancy
Life time
Interval time
Switching rate
From theta transition probability
From alpha transition probability
From low beta transition probability
From high beta transition probability
From background transition probability
Within band states transition probability
Features

Supplementary Figure 5: LFP HMM state prediction of change in contralateral bradykinesia-rigidity without tremor items upon treatment with levodopa. (**A**) Box-and-whisker plot of coefficient of determination (r^2) between predicted and actual percentage improvement in contralateral hemibody bradykinesia-rigidity items of the UPDRS Part III. Prediction was performed using ridge regression which utilized features extracted from different HMM models (Left; compare with left hand HMM result in Fig 6) and thresholding method (right). Each dot represents data from one HMM model (n=56) and one thresholding model (n=23; one for each threshold applied across the four frequency bands). ***: p<0.001. (**B**) Illustrative example of single HMM and thresholding models show correlation between predicted and actual percentage improvement in contralateral hemibody bradykinesia-rigidity items of the UPDRS Part III. Prediction was performed using ridge regression which utilized multiple HMM state features extracted from an example 8-state and 7 lags HMM model (**top**) or a 75th percentile thresholding model (**bottom**) for the different frequency bands. Results of leave-out one cross-validation are presented for the ridge regression model. Each dot represents data from one hemisphere in one subject (n=64). Linear fits and 95% confidence limits are shown as straight lines. Negative changes represent % reductions in UPDRS score after medication administration.

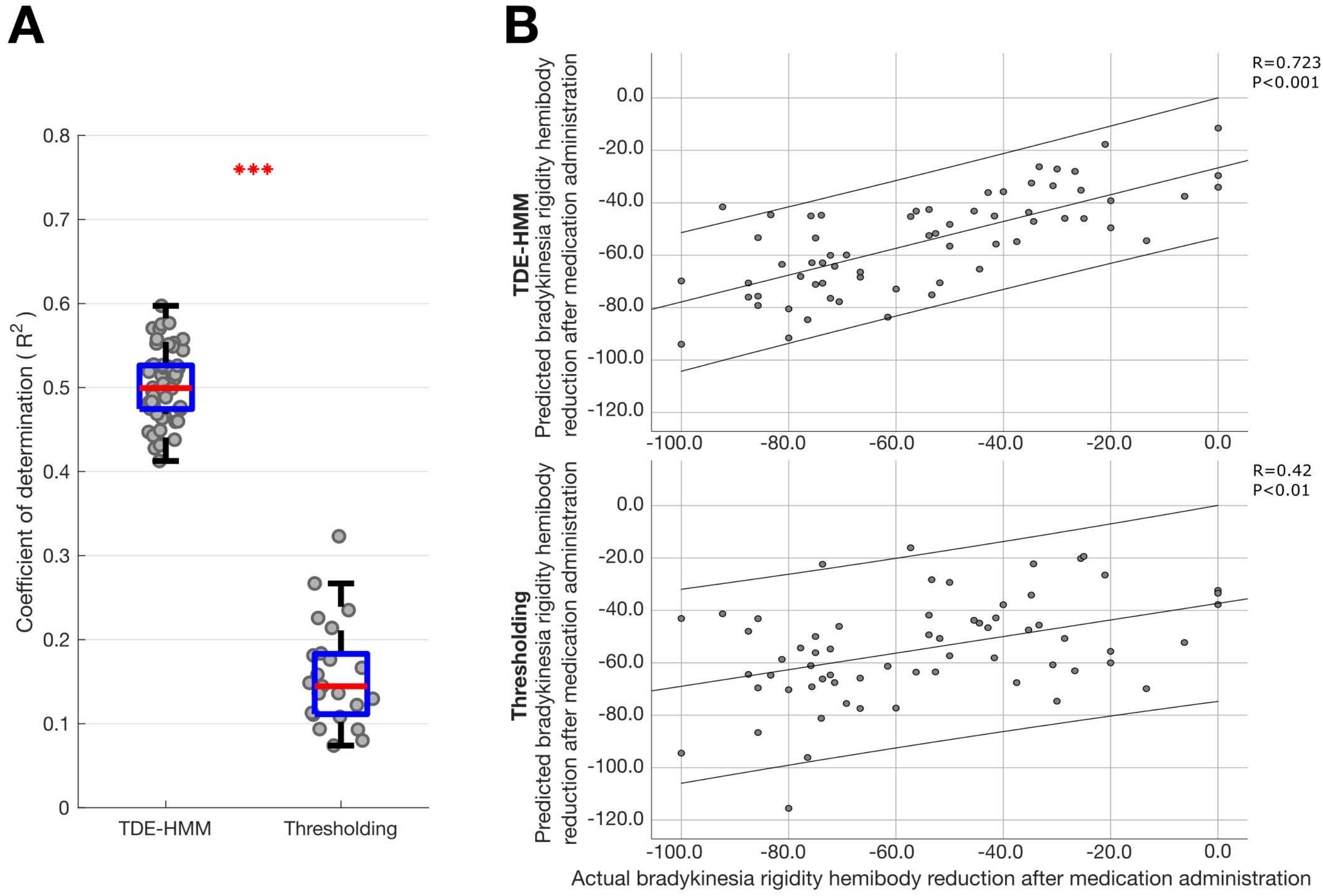
A
B
Coefficient of determination (R^2)
0.8
0.7
0.6
0.5
0.4
0.3
0.2
0.1
0
***
TDE-HMM
Thresholding
R=0.723
P<0.001
R=0.42
P<0.01
TDE-HMM
Thresholding
Predicted bradykinesia rigidity hemibody reduction after medication administration
0.0
-20.0
-40.0
-60.0
-80.0
-100.0
-120.0
Actual bradykinesia rigidity hemibody reduction after medication administration

Supplementary Figure 6: Box-and-whisker plot of LFP HMM state prediction of change in contralateral tremor upon treatment with levodopa. Coefficient of determination (r^2) between predicted and actual percentage improvement in contralateral hemibody tremor items of the UPDRS Part III. Prediction was performed using ridge regression which utilized features extracted from different HMM (**left**) and thresholding models (**right)**; compare with left and right hand HMM result in Supplementary Figure 5A, respectively) . Each dot represents data from one HMM model (n=56) and one thresholding model (n=23; different threshold levels 55-99). ***: $p<0.001$.

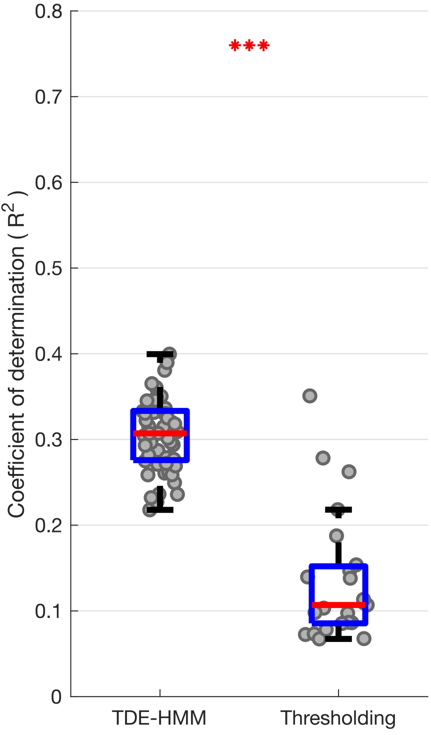

0.8
0.7
0.6
0.5
0.4
0.3
0.2
0.1
0
Coefficient of determination (R²)
***
TDE-HMM
Thresholding

Supplementary Figure 7: Normalised ridge regression coefficients for the features used to predict change in contralateral hemibody tremor score across different HMM models. The most significant ($P<0.001$) contributions to the regression models for change in tremor come from medication-induced changes in the relative number of longer bursts in the high beta band, occurrence rate of shorter bursts in the theta band, longer interval time in the low beta band and higher transition probabilities from the alpha to the high beta state, from the low beta to the theta and high beta states, from the high beta states to the theta state, and within detected high beta states. All these contributions were positive, i.e. predicted a greater reduction in tremor. Median normalised regression coefficients across the 56 different HMM models ± SD are shown. *: $p<0.05$, **:$p<0.01$, ***: $p<0.001$.

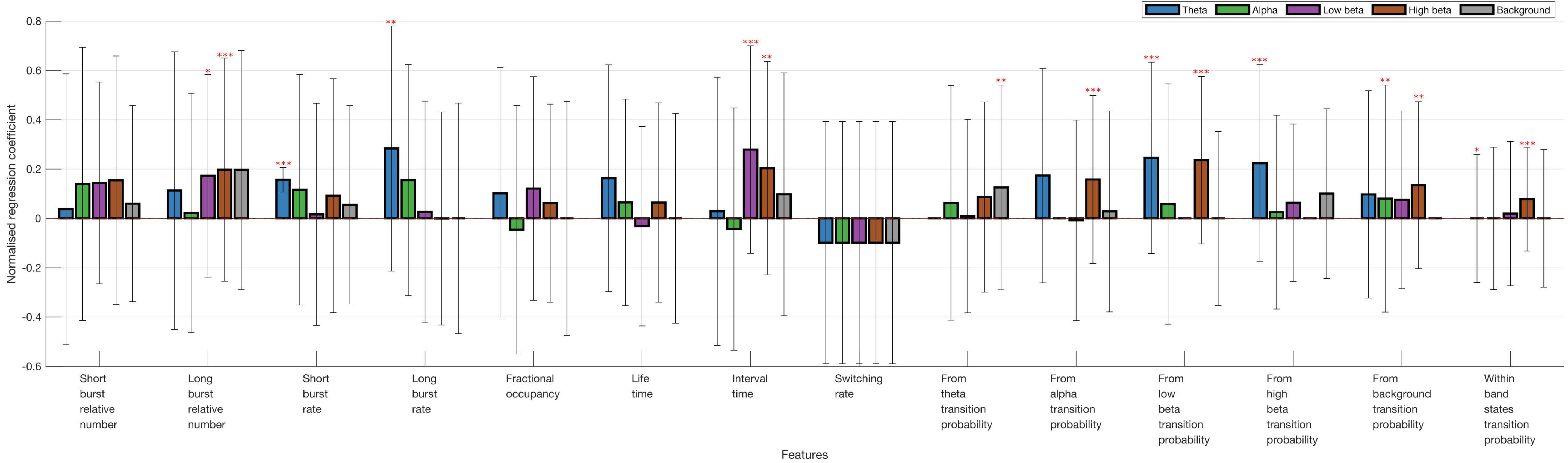

Theta
Alpha
Low beta
High beta
Background
Normalised regression coefficient
0.8
0.6
0.4
0.2
0
-0.2
-0.4
-0.6
Short burst relative number
Long burst relative number
Short burst rate
Long burst rate
Fractional occupancy
Life time
Interval time
Switching rate
From theta transition probability
From alpha transition probability
From low beta transition probability
From high beta transition probability
From background transition probability
Within band states transition probability
Features

Supplementary Figure 8: Box-and-whisker plots of LFP HMM state prediction of change in contralateral hemibody UPDRS score including tremor items upon treatment with levodopa after including states and bands amplitude information (A) and both amplitude and gamma features (B). Coefficient of determination (r^2) between predicted and actual percentage improvement in contralateral hemibody UPDRS score including tremor items. Prediction was performed using ridge regression which utilized features extracted from different HMM models **(left)** and different percentile thresholding models across different frequency bands **(right)**. Each dot represents data from one HMM model (n=56) on the left and one thresholding model (n=23; different threshold levels 55-99) on the right. ***: $p<0.001$.

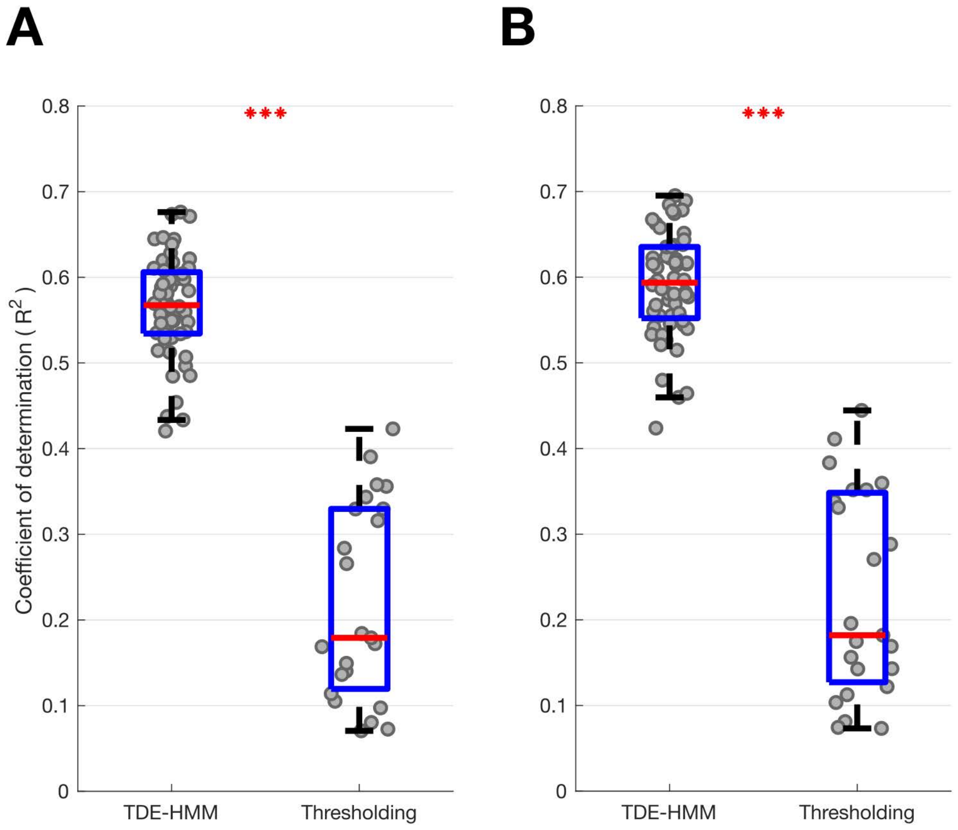

A
B
0.8
0.7
0.6
0.5
0.4
0.3
0.2
0.1
0
Coefficient of determination (R2)
***
TDE-HMM
Thresholding

Supplementary Figure 9: Box-and-whisker plots of LFP HMM state prediction of contralateral bradykinesia-rigidity (A) and contralateral tremor (B) OFF levodopa. Coefficient of determination (r^2) between predicted and actual hemibody items of the UPDRS Part III. Prediction was performed using ridge regression which utilized features extracted from different HMM and thresholding models. Each dot represents data from one HMM model (n=56) and one thresholding model (n=23; different threshold levels 55-99). ***: $p<0.001$.

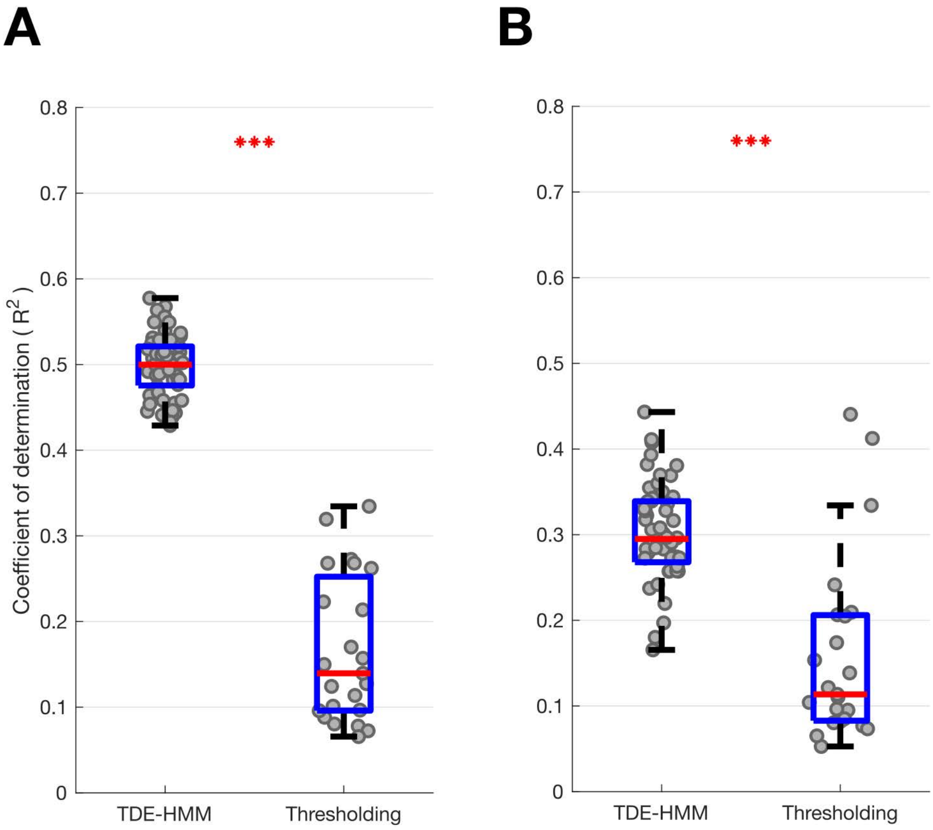
A
B
Coefficient of determination (R2)
0.8
0.7
0.6
0.5
0.4
0.3
0.2
0.1
0
***
TDE-HMM
Thresholding

Supplementary Figure 10: Normalised ridge regression coefficients for the features used to predict contralateral hemibody UPDRS scores including tremor items during OFF medication across different HMM models. The states and features contributing to the regression models are very similar to those contributing to regression models predicting the change in motor impairment with levodopa (see Supplementary Figure 4). Median normalised regression coefficients across the 56 different HMM models ± SD are shown.*: $p<0.05$, **:$p<0.01$, ***: $p<0.001$.

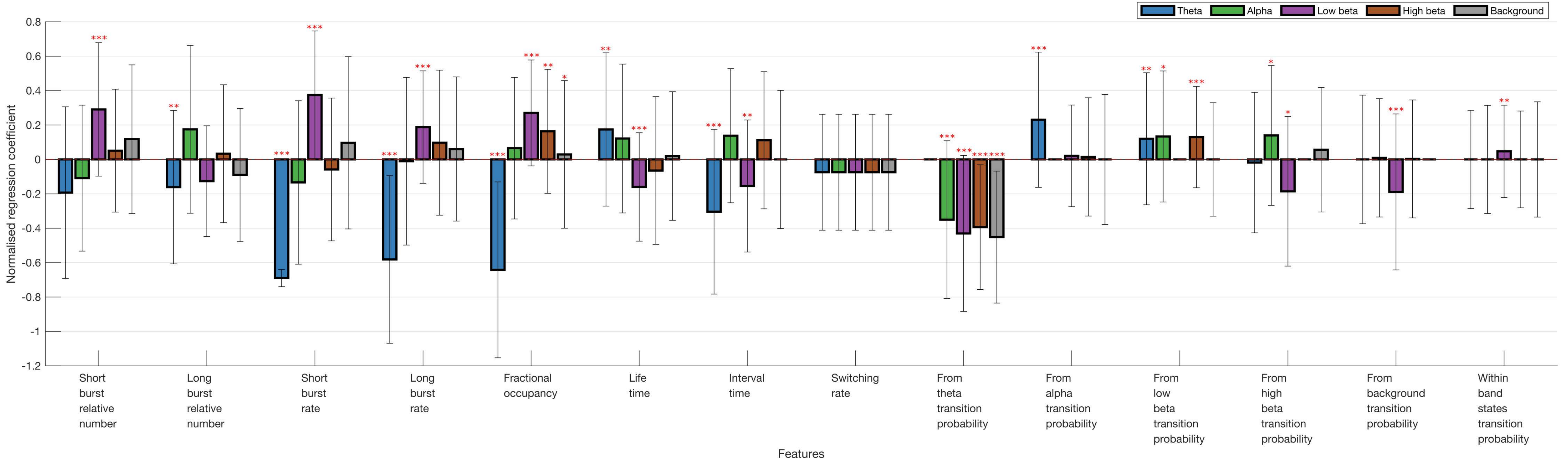

Theta
Alpha
Low beta
High beta
Background
Normalised regression coefficient
0.8
0.6
0.4
0.2
0
-0.2
-0.4
-0.6
-0.8
-1
-1.2
Short burst relative number
Long burst relative number
Short burst rate
Long burst rate
Fractional occupancy
Life time
Interval time
Switching rate
From theta transition probability
From alpha transition probability
From low beta transition probability
From high beta transition probability
From background transition probability
Within band states transition probability
Features

Supplementary Figure 11: Scatterplot of normalised ridge regression coefficients for the features used to predict change in contralateral hemibody UPDRS score including tremor items ON medication and for the features used to predict contralateral hemibody UPDRS scores including tremor items during OFF medication, both across the 56 different HMM models. These tend to lie on a diagonal which means that the LFP features that predict motor impairment OFF medication are those that are modulated by treatment with levodopa to achieve improvement in motor state.

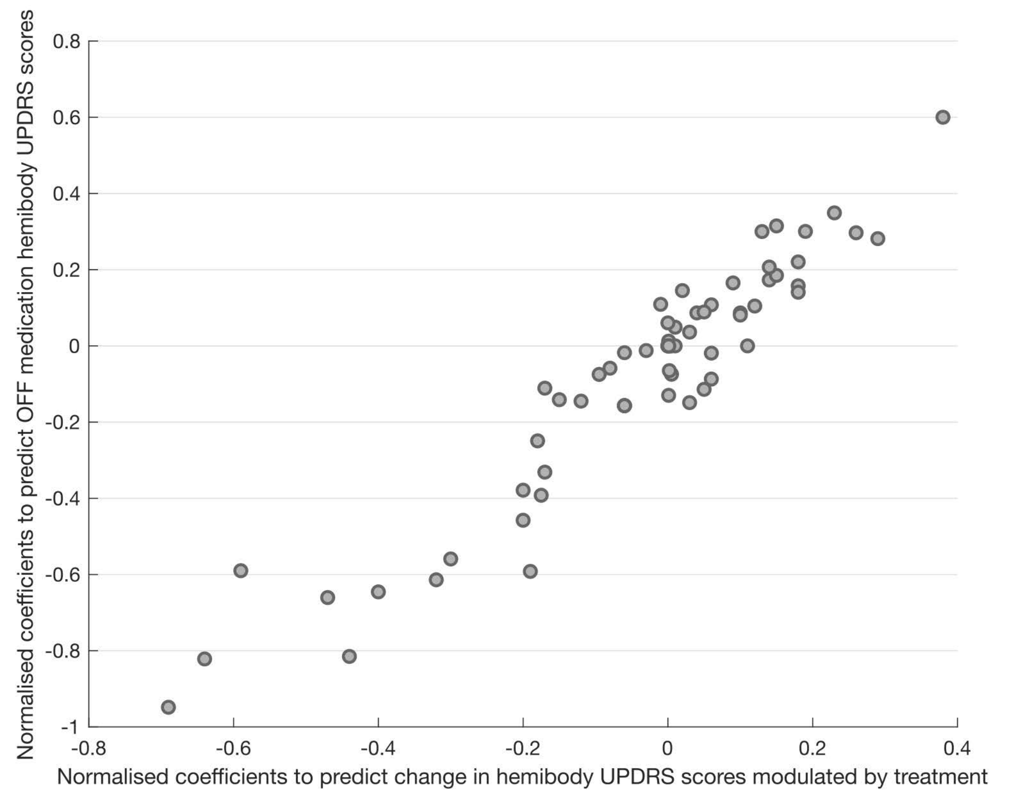
Normalised coefficients to predict OFF medication hemibody UPDRS scores
0.8
0.6
0.4
0.2
0
-0.2
-0.4
-0.6
-0.8
-1
-0.8
-0.6
-0.4
-0.2
0
0.2
0.4
Normalised coefficients to predict change in hemibody UPDRS scores modulated by treatment

Supplementary Figure 12: Box-and-whisker plots of LFP HMM state prediction of contralateral hemibody UPDRS score including tremor items after including states and bands amplitude information (A) and both amplitude and gamma features (B) OFF levadopa. Coefficient of determination (r^2) between predicted and actual contralateral hemibody UPDRS score, including tremor items. Prediction was performed using ridge regression which utilized features extracted from different HMM models **(left)** and different percentile thresholding models across different frequency bands **(right)**. Each dot represents data from one HMM model (n=56) on the left and one thresholding model (n=23; different threshold levels 55-99) on the right. ***: $p<0.001$.

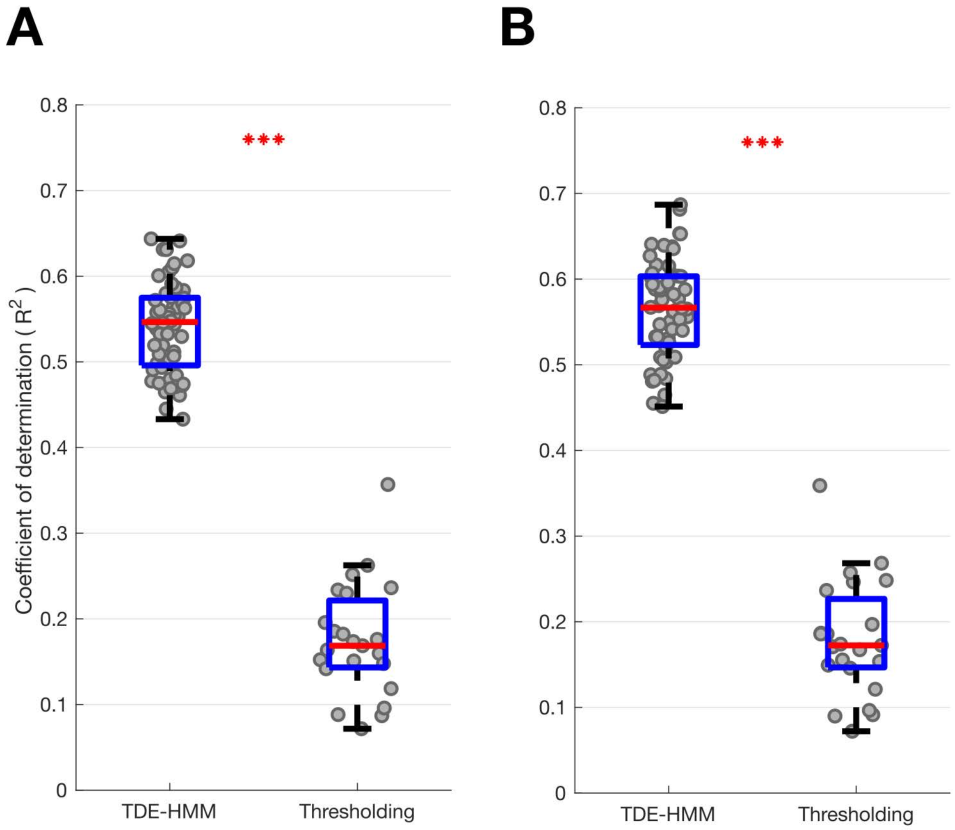
A
B
Coefficient of determination (R2)
0.8
0.7
0.6
0.5
0.4
0.3
0.2
0.1
0
***
TDE-HMM
Thresholding
